# From bound states to quantum spin models: chiral coherent dynamics in topological photonic rings

**DOI:** 10.1515/nanoph-2025-0473

**Published:** 2025-11-25

**Authors:** Fatemeh Davoodi

**Affiliations:** Nanoscale Magnetic Materials, Institute of Materials Science, Kiel University, 24143 Kiel, Germany; Kiel Nano, Surface and Interface Science KiNSIS, Christian Albrechts University, Kiel, Germany

**Keywords:** Chiral quantum optics, Su-Schrieffer-Heeger lattice, Topological photonics, Physics-informed deep learning

## Abstract

Topological photonic systems offer a robust platform for guiding light in the presence of disorder, but their interplay with quantum emitters remains a frontier for realizing strongly correlated quantum states. Here, we explore a ring-shaped Su-Schrieffer-Heeger (SSH) photonic lattice interfaced with multiple quantum emitters to control topologically protected chiral quantum dynamics. Using a full microscopic model that includes cascaded Lindblad dynamics and chiral emitter-bath couplings, we reveal how the topology of the bath mediates nonreciprocal, long-range interactions between emitters. These interactions lead to rich many-body spin phenomena, including robust coherence, directional energy transfer, captured by an effective spin Hamiltonian derived from the system’s topology. We show that topological bound states enable unidirectional emission, protect coherence against dissipation, and imprint nontrivial entanglement and mutual information patterns among the emitters. In particular, we showed that under circularly polarized excitation, the emitters not only inherit spin angular momentum from the field but also serve as transducers that coherently launch the spin-orbit-coupled topological photonic modes into the far field. Our results establish a direct bridge between topological photonic baths and emergent quantum magnetism, positioning this architecture as a promising testbed for studying chiral quantum optics, topologically protected entangled states, and long-range quantum coherence.

## 1 Introduction

**Figure 1:**
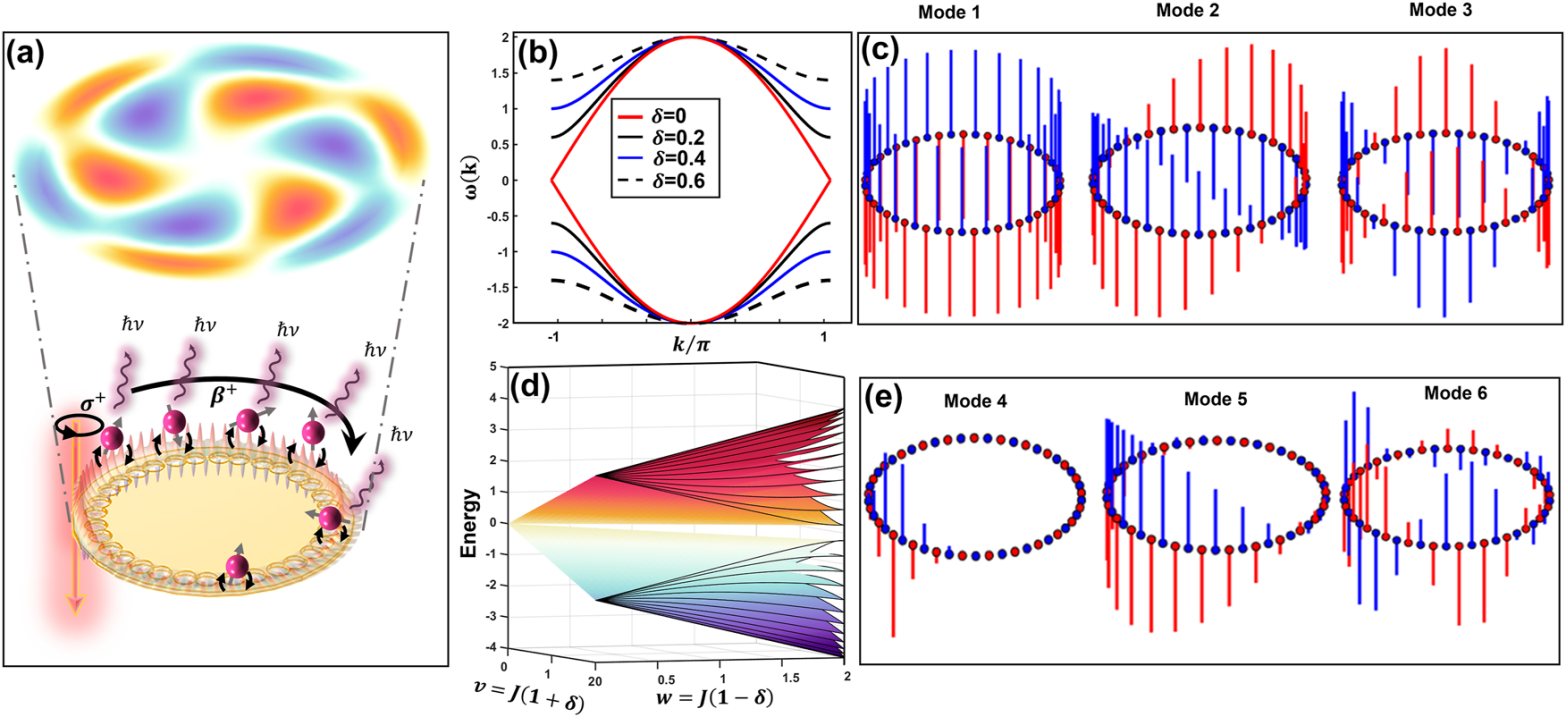
Topological photonic SSH ring with chiral emitter coupling and mode analysis. (a) Schematic of a dimerized SSH ring supporting excited charily at topological photonic modes using circularly polarized pump. The topological modes in SSH chain launching unidirectional (chiral) excitation around the ring, interact and excite the quantum emitters (gold nano sphere) in chiral way by the coupling coefficient *β* ^+^. The emitted field inherits the vortex profile of the SSH eigenmode, coherently transferred to the far field via Purcell-enhanced dipole emission. (b) Photonic band structure of the SSH model for several values of the dimerization parameter *δ*. Increasing *δ* opens a topological bandgap around *ω* = 0, isolating in-gap edge states (black dashed lines). (c–e) Eigenmode profiles of a finite SSH ring. Vertical bars show the field amplitudes on sublattice sites A (red) and B (blue). (c) Modes 1–3 exhibit bulk-like symmetric delocalization. (e) Modes 4–6 display staggered or asymmetric localization, reflecting consistent with localized or chiral behaviour.

Preserving quantum coherence in complex photonic environments is a central challenge in quantum science [1], [2], key advances in single-photon sources [3], [4], quantum communication, and beyond [5], [6], [7], [8]. Decoherence, often attributed to losses, scattering, or disorder, can instead be suppressed, or even harnessed, through careful engineering of the environment itself [9], [10]. Topological photonics offers a powerful framework for such control: exploiting symmetry, interference, and topological invariants, it enables robust light–matter interactions protected against disorder [11], [12]. Platforms inspired by the Su–Schrieffer–Heeger (SSH) model provide an ideal architecture to explore this paradigm [13], [14], [15]. These systems support edge-localized photonic modes with chiral propagation, immune to backscattering even in the presence of fabrication imperfections or material losses; material loss will attenuate the mode but does not, by itself, break the topological protection as long as the bandgap remains open [12], [16], [17], [18], [19]. When coupled to quantum emitters, such topological baths mediate unconventional dynamics: unidirectional coupling, non-Markovian evolution, fractional decay, and photonic bound states even in the weak-coupling regime [20], [21], [22], [23]. The structured environment can control and give rise to long-range, phase-coherent interactions between emitters, governed by an emergent many-emitter effective spin Hamiltonian. These interactions, encoded in the Green’s tensor of the photonic bath, inherit its range and chirality [24], enabling nontrivial [22] many-body effects such as unidirectional superradiance, quantum spin coherence, and topological phases including a double Néel state. We design and analyze a realistic nanophotonic SSH ring in a 40-nm gold film (32 nanoholes, 16 unit cells) (Figure 1a). A physics-informed deep-learning framework yields a dimerized geometry that supports vortex-like topological edge modes with azimuthal phase winding and transverse spin-orbit coupling (SOC) [25], [26]. Circularly polarized light carrying orbital angular momentum (OAM) selectively launches unidirectional edge modes. Gold nanospheres (60 nm) at selected nanoholes act as quantized dipolar emitters [27], [28], coupling spin-selectively to co-propagating edge modes via spin-OAM locking. The platform provides a chiral quantum interface that enables directional excitation and vortex-beam emission, uniting topological photonics, quantum spin physics, and quantized plasmonics.

## 2 SSH bath Hamiltonian in a ring geometry

The SSH lattice comprises (A/B) sublattice per unit cell with alternating intra-cell (*ν*) and inter-cell (*w*) coupling (Supplementary Figures S_1_ and S_2_). The two bands are symmetric about zero due to chiral sublattice symmetry; a bandgap opens around zero and closes at *ν* = *w* (Figure 1b and d). In the SSH ring, two topologically distinct phases arise depending on $\left|\nu /w\right|$ [29]. The magnitude of the dimerization parameter $\delta =\left(\nu -w\right)/\left(\nu +w\right)$ controls gap opening; its sign sets the chirality of edge-like gap modes and determines whether clockwise or counter-clockwise propagation is excited for a given sublattice and unit-cell origin (Figure 1c and e).

The topology is captured by the winding number *ζ* (Eq. (4)), which counts how many times the Bloch vector winds around the origin across the Brillouin zone [30]:

(1) $$\zeta =\frac{1}{2\pi }\overset{\pi }{\underset{-\pi }{\int }}\frac{\text{d}}{\text{d}k}\arg\left(f\left(k\right)\right)\text{d}k$$

Here in SSH ring shape, it counts how many times the vector $f\left(k\right)=-J\left[\left(1+\delta \right)+\left(1-\delta \right){\text{e}}^{-\text{i}k}\right]$ winds around the origin in the complex plan as $k\in \left[-\pi ,\pi \right]$ . In an SSH chain in aging geometry with one domain wall with nearest neighbour hopping only, *ζ* = ±1 introduces topologically nontrivial edge states and *ζ* = 0 represents the trivial edge states. Although a closed ring has no physical edges, *ζ* manifests in the sublattice phase structure and in directional emitter-bath coupling. Superlattice modulations or domain-wall interference increase winding, yielding multiple edge modes (Figure S3) and higher-order topological features [26] (see Supplementary Section I). The manifestation of positive and negative winding numbers (*ζ*) becomes apparent upon analysis of the orbital angular momentum associated with these modes. Detailed visualizations of the orbital angular momentum pertaining to both of these topologically nontrivial modes will be discussed later.

## 3 Quantum emitters and interaction with the bath

By introducing *N*_*e*_ two-level quantum emitters (QEs) embedded in or near specific sublattice sites (A and B), each emitter locally couples to the photonic mode of that site. Let $\left|g\right\rangle$ and $\left|e\right\rangle$ be the ground and excited states of a QE, with transition frequency *ω*_*e*_. We define the detuning Δ = *ω*_*e*_ − *ω*_*a*_ from the mid-gap frequency. The free Hamiltonian for the emitters is simply [20]:

(2) $$H_{S}=\Delta \overset{N_{e}}{\underset{m=1}{\sum }}\sigma_{m}^{ee}$$

where $\sigma_{m}^{ee}\left|e_{m}\right\rangle \left\langle e\right|$ is the projector onto the excited state of emitter *m*.

When the emitters interact with the structured photonic bath, one can derive an effective open-system master equation for the emitters alone by tracing out the photonic modes. In the Born–Markov approximation, the reduced density matrix *ρ* of the emitters obeys [31], [32]:

(3) $$\begin{array}{ll} \rho & =-ı\left[H_{S}^{\;\cdot }+H_{LS},\rho \right]+\underset{m,n}{\sum }\frac{\Gamma_{mn}^{ab}}{2}\left(2\sigma_{n}^{ge}\rho \sigma_{m}^{eg}-\sigma_{m}^{eg}\sigma_{n}^{ge}\rho \right.\\ & \;\left.-\rho \sigma_{m}^{eg}\sigma_{n}^{ge}\right) \end{array}$$

**Figure 2:**
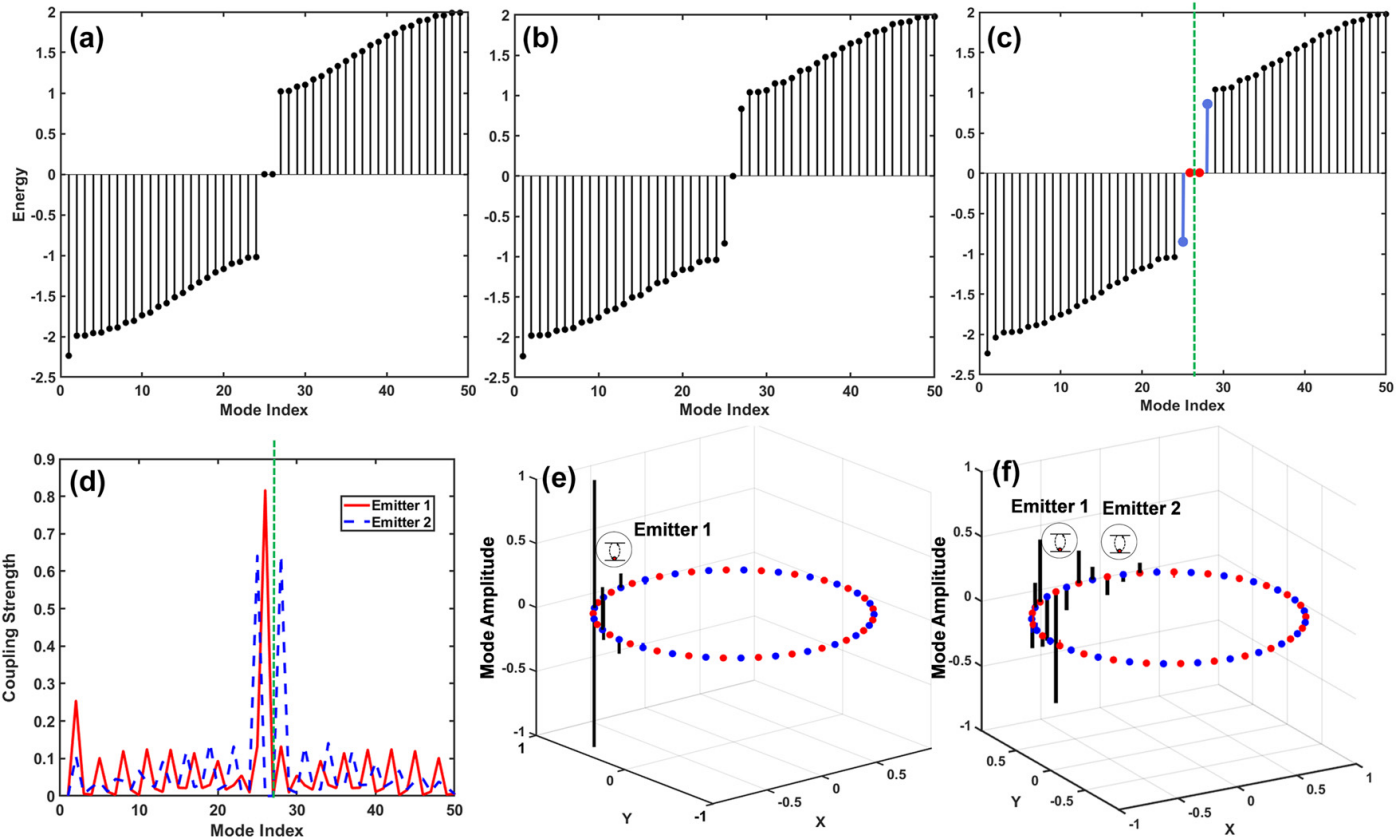
Chiral mode structure and emitter coupling in a topological SSH ring. (a–c) Energy spectra of the SSH ring with *N* = 48 lattice sites (24 dimers), showing the emergence of in-gap states due to emitter coupling. (a) The bare SSH ring with *δ* = 0.5 exhibits a clear bandgap and topological edge modes under periodic boundary conditions. (b) Introducing a single emitter perturbs the system and induces a localized bound state near mid-gap. (c) With two emitters, the in-gap structure becomes more complex, exhibiting mode hybridization as the emitters couple coherently through the bath. The mode index spans all eigenmodes sorted by energy. (d) Mode-resolved coupling strengths ${\left|\beta^{+}\left(k\right)\right|}^{2}$ for each emitter, showing asymmetric spectral overlap. Emitter 1 (red solid) couples more strongly to mid-gap modes than emitter 2 (blue dashed), illustrating chiral coupling governed by the SSH bath topology and spatial mode structure. The topological mid-gap hybrid modes are highlighted in (c) (red and blue markers) and its mode index is indicated by the green dashed line. The same index is marked in (d), showing that both emitter 1 (red) and emitter 2 (blue) couple strongly to this gap mode. The different peak amplitudes for emitter 1 and emitter 2 illustrate the chiral, directional coupling between the edge mode and the two emitters. (e, f) Mode amplitude distributions around the ring for configurations with one (e) and two (f) emitters. Red and blue dots denote amplitudes on sublattices A and B, respectively. Arrows indicate direction of topological energy flow, aligned with the sign of *δ* and the *β* ^+^ mediated unidirectional emission. The Lamb shift, originating from virtual photon exchange via SSH eigenmodes, shifts the effective energies of the emitters and modifies the collective dynamics.

This equation includes both coherent Lamb shifts (through an effective Hamiltonian $H_{LS}=\sum_{m\cdot n}J_{mn}^{ab}\sigma_{m}^{eg}\sigma_{n}^{ge}+\text{H}.\text{c}$ ., which is responsible for coherent interactions mediated by the bath, and dissipative decay terms between emitter *n* and *m*) and $\Gamma_{mn}^{ab}$ , dissipation. Here $a,b\in \left\{A,B\right\}$ label the sublattices of emitters *m*, *n*. For unidirectional baths, the reduced emitter master equation acquires a cascaded Lindblad structure with nonreciprocal couplings [23], [32]:

(4) $$\begin{array}{ll} \rho & =-ı\left[\dot{H_{S}}+H_{LS},\rho \right]+\underset{m<n}{\sum }\Gamma_{mn}\left(2\sigma_{n}^{-}\rho \sigma_{m}^{+}-\sigma_{m}^{+}\sigma_{n}^{-}\rho \right.\\ & \;\left.-\rho \sigma_{m}^{+}\sigma_{n}^{-}\right) \end{array}$$

where $H_{LS}=\sum_{i}\Delta_{i}\sigma_{i}^{+}\sigma_{i}^{-}$ is free emitter Hamiltonian and $\sigma_{i}^{+}=\left|e\right\rangle \left\langle g\right|$ , $\sigma_{i}^{-}=\left|g\right\rangle \left\langle e\right|$ are raising/lowering operators. As shown in Figure 2, by introducing one emitter near the two topological edge states of the ring (Figure 2a), coupling to the edge mode, breaks the perfect bath symmetry and creates an avoided crossing leads to splitting the original edge state (Figure 2b), if the second emitters is introduced, effectively forming a cascaded chain through the photonic bath Figure 2c. The dominant contribution to topological mode excitation of emitter 1 and weaker and shifted response of emitter 2, is because emitter 2 receives energy from emitter 1, but cannot influence it back. The asymmetry in coupling strengths (Figure 2d) which is due to the chirality and directional propagation from emitter 1 to emitter 2 (Figure 2e and f) is the direct evidence of chiral, unidirectional dynamics. This non-Hermitian extension and nonreciprocal coupling leading to asymmetric energy flow and a cascade structure in eigenvalues which predicted by rich open-system cascaded Lindblad dynamics.

## 4 Photonic bound states and chirality

If a QE’s frequency lies in a bandgap, decay into propagating modes is suppressed and a bound state (BS) forms, localized around the emitter. The BS wavefunction is [33]:

(5) $$\left|\Psi \left(t\right)\right\rangle =C_{e}\left(t\right)\left|e;\text{Vac}\right\rangle +\underset{j,\alpha \in \left\{A,B\right\}}{\sum }C_{e,\alpha }\left(t\right)\left|g;1_{j,\alpha }\right\rangle$$

where $C_{e}\left(t\right)$ equal to the long-time excited-state amplitude. In the single-excitation sector, the state takes the form of Eq. (5), and its energy is given by the self-consistency condition Eq. (6), with up to three solutions (lower/middle/upper gaps) owing to divergences at band edges [34]:

(6) $$E_{\text{BS}}=\Delta +\underset{ee}{\sum }\left(E_{\text{BS}}\right)$$

The BS coefficients (Eq. (5)) yield a fractional decay: the long-time excited-state population equals ${\left|C_{e}\right|}^{2}$ , which is finite at mid-gap $\left(\Delta =0\right)$ . The localization length scales as $L_{\text{BS}}=-\frac{1}{\ln\left|\frac{1-\delta }{1+\delta }\right|}$ , increasing as the gap closes. A striking feature is chiral localization: at Δ = 0, the BS is confined to one sublattice on one side of the emitter ( $C_{j,A}=0,C_{j,B}=gC_{e}{\left(-1\right)}^{j}\frac{1}{J\left(1+\delta \right)}{\left(\frac{1-\delta }{1+\delta }\right)}^{j}\;\;\text{for}\;j\geq 0$ ), with the occupied sublattice and direction set by sign of *δ*. If *L*_BS_ approaches *N* (when the gap nearly closes), the bound state’s evanescent tail can wrap around the ring and meet the “back” of the emitter. In that regime the strictly one-sided solution is not unique, the clockwise and counter clockwise decaying solutions hybridize into two standing-wave modes (one symmetric, one antisymmetric). However, for a moderate ring size (for example, 16 cells) and a reasonably sized gap, the bound state is effectively chiral and confined to one side of the QE (for more information see Supplementary Information Section III). While we use the minimal nearest-neighbour SSH bath Hamiltonian to define the topological charge and the chiral gap, we verified that adding moderate further-neighbour couplings only renormalizes the gap and the bound-state localization length *L*_BS_ without altering winding number *ζ* as long as the gap remains open.

## 5 Emitter–emitter interactions and directionality

With multiple emitters in a gap, exchange of virtual photons via BSs generates a purely coherent Hamiltonian in the Markovian limit [35], [36]:

(7) $$H_{\text{eff}}=J_{mn}^{ab}\left(\sigma_{m}^{eg}\sigma_{mn}^{eg}+\text{H.C.}\right)$$

with exponentially decaying $J_{mn}^{ab}=Re\sum_{mn}^{ab}\left(\omega_{e}\right)$ . The sign and sublattice dependence of $J_{mn}^{ab}$ encode topology: for mid-gap $\left|\Delta \right|=0$ , same-sublattice couplings cancel while cross-sublattice couplings dominate and alternate in sign with distance. Flipping *δ* reverses the sign of same-sublattice $J_{mn}^{ab}$ but leaves cross-sublattice couplings unchanged. In a ring, geometry makes these couplings chiral. For emitters on A at cell *n* and B at *m*, Eq. (7) gives $J_{nm}\propto e^{-d/L_{\text{BS}}}$ only when *m* is ahead of *n* in the direction set by *δ*; the reverse orientation is exponentially suppressed. This non-reciprocal behaviour is a direct consequence of the one-sided BS: emitter 1 can send a virtual photon only into its forward sublattice. Finite rings yield exponentially small “forbidden” couplings except when emitters are nearly opposite, where forward/backward paths merge.

**Figure 3:**
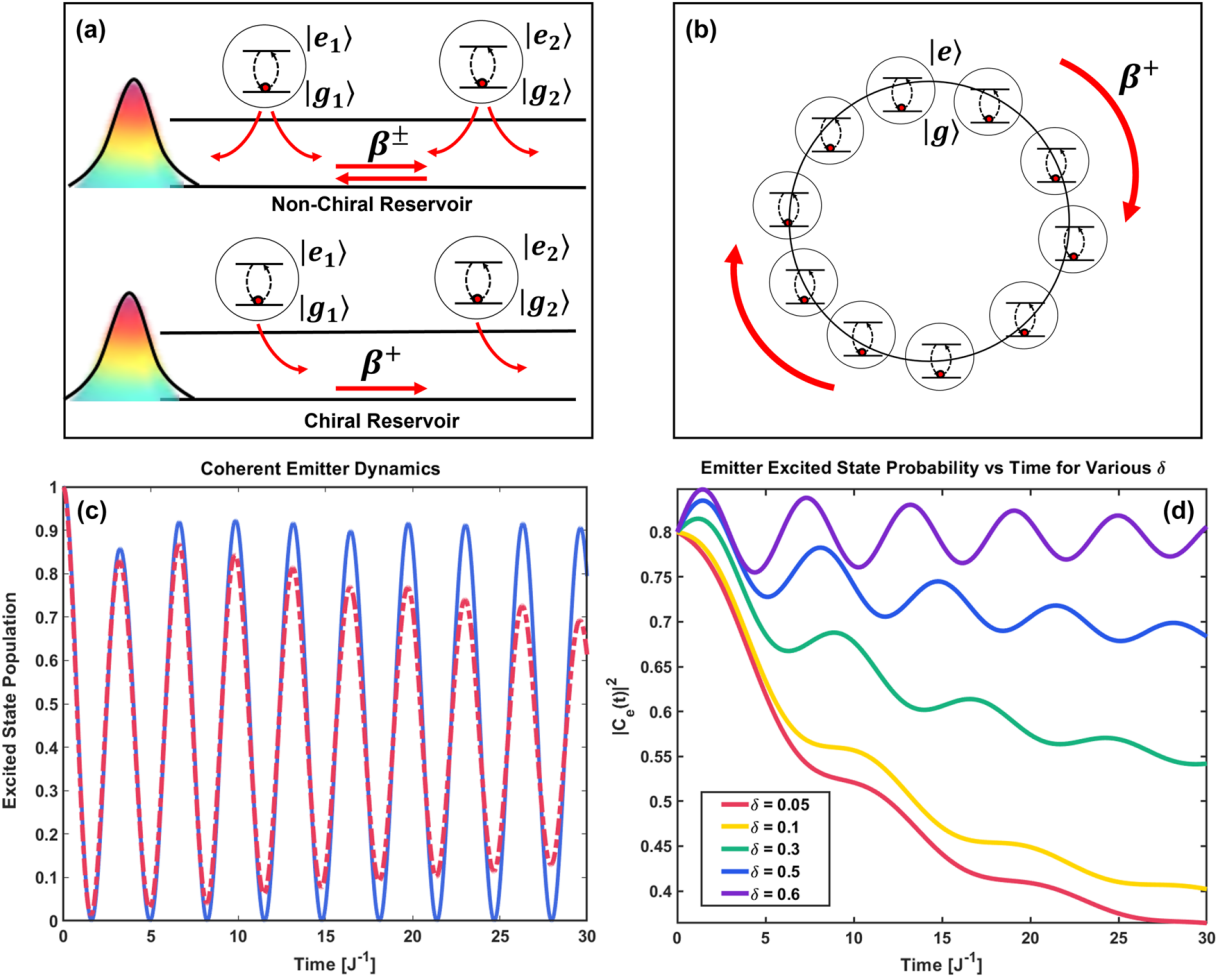
Topology-enabled chiral light–matter interactions and coherent dynamics in structured reservoirs. (a) Schematic comparison between a non-chiral (top) and chiral (bottom) reservoir. In the non-chiral case, symmetric emission leads to bidirectional photon exchange between emitters via coupling constants *β* ^±^. In contrast, the chiral reservoir supports unidirectional energy flow (*β* ^+^), enabling nonreciprocal emitter-emitter inter actions. (b) Chiral propagation around a topological SSH ring with embedded emitters. The directionality of excitation transfer is governed by the sign of the dimerization *δ*, with *β* ^+^ enforcing clockwise photon-mediated coupling. (c) Coherent emitter dynamics in the presence (solid blue) and absence (dashed red) of dissipation. Even in a dissipative photonic bath, topological protection preserves long-lived oscillations in the excited-state population, indicating robust coherence. (d) Time evolution of the emitter excited-state probability ${\left|C_{e}\left(t\right)\right|}^{2}$ for several values of the dimerization parameter *δ*. As *δ* increases, opening a stronger topological bandgap, the system exhibits enhanced coherence, slower decay, and saturation at higher population plateaus. This demonstrates the role of bath topology in preserving emitter coherence and suppressing decoherence.

One can quantify nonreciprocity via the chiral coupling efficiency [37]:

(8) $$\beta_{mn}=\frac{{\left|\underset{mn}{\sum }\right|}^{2}}{{\left|\underset{mn}{\sum }\right|}^{2}+\xi^{2}}=\frac{J_{mn}^{2}+{\left(\Gamma_{mn}\;/2\right)}^{2}}{J_{mn}^{2}+{\left(\Gamma_{mn}\;/2\right)}^{2}+\xi^{2}}$$

which remains high in our topological bath even with moderate loss. Figure 3 shows that coherence oscillations survive dissipation due to symmetry and topological protection. Simulations with six randomly placed emitters (Figure 3d) confirm direction-dependent coupling and robust localization mediated by BSs, qualitatively distinct from conventional (non-topological) reservoirs.

**Figure 4:**
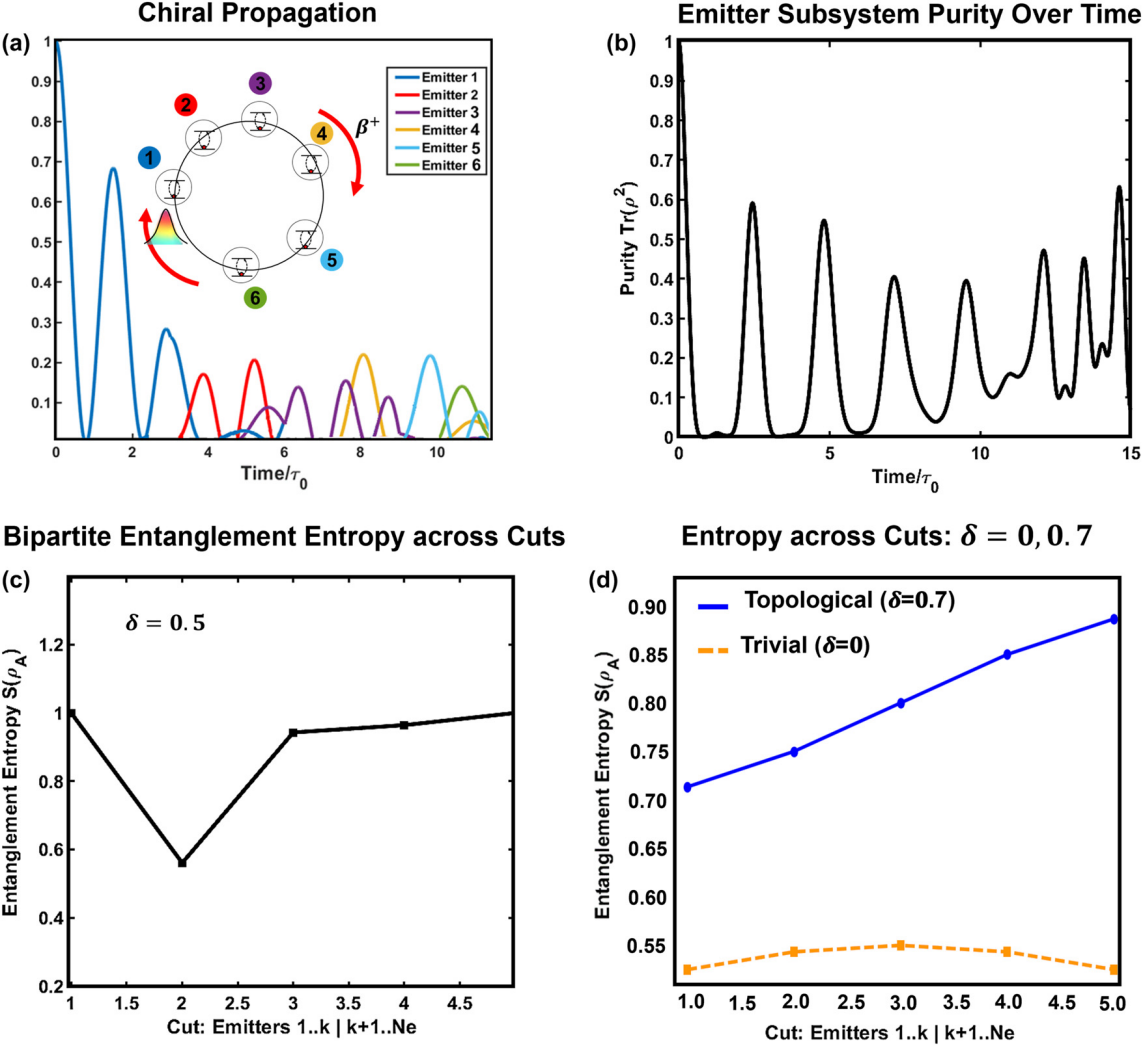
Chiral energy transfer and entanglement growth in a topological emitter array. (a) Time-resolved excitation dynamics of six emitters coupled in a chiral photonic SSH ring. An initial excitation on emitter 1 propagates unidirectionally (*β* ^+^) through the ring, sequentially exciting emitters 2 through 6 in a coherent cascade, demonstrating robust topological chiral transport. (b) Subsystem purity Tr(*ρ* ^2^) of the emitter array over time. The recurring dips in purity indicate entanglement generation and coherent mixing with the surrounding emitters, reflecting non-Markovian dynamics governed by the structured bath. (c) Bipartite von Neumann entanglement entropy $S\left(\rho_{A}\right)$ across spatial cuts of the emitter chain for $\left|\delta \right|=0.5$ . The entropy dip at the central cut reflects a double Néel-type spin structure, consistent with alternating sublattices and topology-induced correlations. (d) Comparison of entanglement entropy between topological ( $\left|\delta \right|=0.7$ ) and trivial ( $\left|\delta \right|=0$ ) regimes. The topological configuration supports significantly stronger entanglement across all bipartitions, revealing long-range quantum correlations mediated by bound photonic edge modes.

As the excitation moves between emitters, the reduced dynamics of the emitter subsystem becomes highly non-Markovian (see Figure 4a). To quantify the coherence of the emitter system, we compute the purity of the reduced density matrix [38]:

(9) $$\text{Purity}\left(t\right)=\mathrm{Tr}\left[\rho_{e}{\left(t\right)}^{2}\right]$$

where $\rho_{e}\left(t\right)$ is obtained by tracing out the photonic bath degrees of freedom. A purity close to 1 indicates low entanglement with the bath and high coherence, while a lower purity reflects decoherence and mixedness due to system-bath entanglement. As demonstrated in Figure 4b, the purity of the emitter subsystem exhibits sharp oscillations during this process, signalling strong non-Markovian dynamics and revivals of quantum coherence. These fluctuations indicate transient entanglement as the excitation rotates around the ring, periodically localizing and delocalizing among the emitters.

The degree of intra-emitter quantum entanglement in such a system can be characterize using the bipartite von Neumann entropy. At each time slice or in the ground state, we partition the emitter array into two contiguous subsystems A and B (emitters 1…*k* and *k* + 1…*N*_*e*_), compute the reduced density matrix $\rho_{A}=\text{T}{\text{r}}_{B}\left[\rho_{e}\right]$ , and evaluate [39]:

(10) $$S\left(\rho_{A}\right)=-\mathrm{Tr}\left[\rho_{A}\text{lo}{\text{g}}_{2}\rho_{A}\right]$$

This entanglement entropy reflects quantum correlations between emitter partitions. As shown in Figure 4c and d, we observe that $S\left(\rho_{A}\right)$ increases with subsystem size and is significantly enhanced in topological phases ( $\left|\delta \right|=0.5,0.7$ ) compared to the trivial case ( $\left|\delta \right|=0$ ). The entropy distribution is also asymmetric, revealing the effect of chiral propagation and directional information flow. In Figure 4, the weaker entanglement weak near position 2 is because of sublattice mismatch between emitter 2 and emitter3 which leads to weaker inter-part correlations with the neighbours. This dip is not appeared in Figure 4d, this is because $\left|\delta \right|=0.7$ and bound states and virtual photon exchange delocalize correlations across the whole emitter chain, leading to smooth, collective entanglement growth. That is, each emitter becomes correlated not just with its neighbour, but with a wider neighbourhood, evidence of topological baths with extended interaction kernels *J*_*mn*_. Thus, no single bipartition sees a weak link strong enough to suppress $S\left(\rho_{A}\right)$ in a noticeable way. The correlation network is denser in comparison to $\left|\delta \right|=0.5$ which means topological baths generate long-range coherent correlations, smoothing out entropy across cuts. On the other hand, the trivial case ( $\left|\delta \right|=0$ ) remains nearly flat and low, reflecting little-to-no quantum correlation across cuts. The dynamics of purity and entanglement entropy confirm that the topological SSH ring bath mediates not only coherent excitation transfer, but also facilitates long-range quantum correlations among the emitters due to the additional symmetry. The many emitters long range coupling leads to effective spin model to capture the collective effects and enters a regime dominated by bound-state-mediated interactions. Such bound states are chiral, exponentially localized, and exhibit asymmetric wavefunctions that propagate unidirectionally around the ring, governed by the sign of the dimerization parameter *δ*.

## 6 Many-emitter effective spin model

Tracing out the photonic bath yields an effective *spin-*_*1/2*_ *model* for the emitters. In the presence of a laser pump which introduces a chemical potential *μ* term in the effective spin Hamiltonian, we can control the average excitation density across the emitter array [8]:

(11) $$\begin{array}{ll} H_{\text{spin}} & =\underset{m,n}{\sum }J_{mn}^{AB}\left(\sigma_{m,A}^{eg}\sigma_{n,B}^{eg}+\text{H.C.}\right)\\ & \;-\frac{\mu }{2}\underset{m}{\sum }\left(\sigma_{m,A}^{z}+\sigma_{m,B}^{z}\right) \end{array}$$

Here $\sigma_{m,A}^{z}=\left|e_{\left.\;,\rangle ,m,A\langle \;\right.}\right.\left.e\right|-\left|g_{\;\rangle m,A\langle \;}\right.\left.g\right|$ is the Pauli-*Z* operator for the emitter on site $\left(m,A\right)$ (and similarly for $\left(m,B\right)$ ). The coupling coefficients $J_{mn}^{AB}$ are derived from the real part of the bath’s Green’s function evaluated at the emitter frequency (see Eq. (4)), which captures the directionality, range, and chirality of the underlying photonic bath. The spin Hamiltonian (11) is bipartite (spins on A only interact with spins on B) and inherits a directionality from the sign of *δ*: each A–B pair interacts only if one is clockwise of the other (for *δ* > 0) or only if one is counter-clockwise of the other (for *δ* < 0). Moreover, the interaction range can be tuned from short to infinite by adjusting $\left|\delta \right|$ .

**Figure 5:**
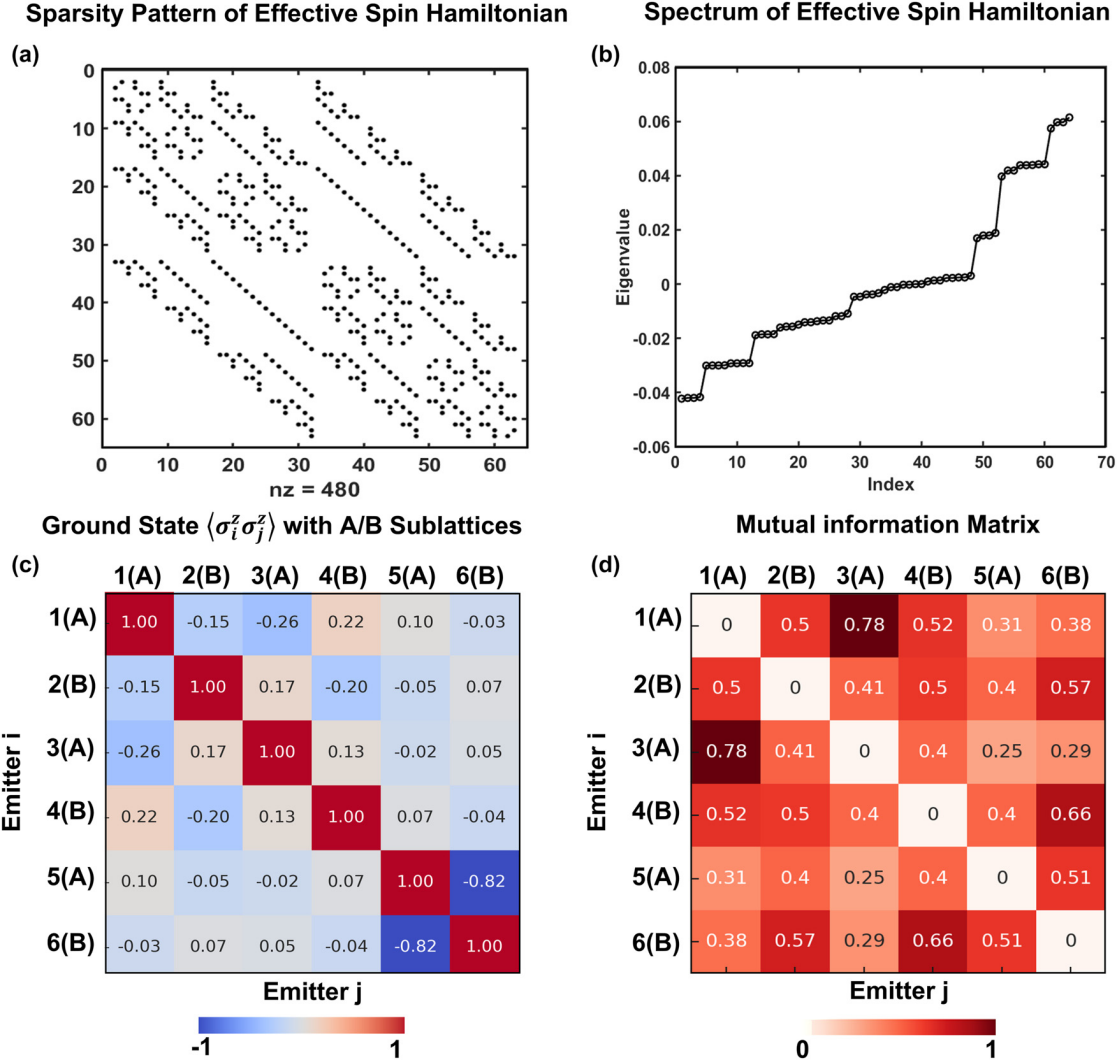
Correlation structure and spectral properties of the many-emitter effective spin Hamiltonian. (a) Sparsity pattern of the effective spin Hamiltonian derived from integrating out the chiral SSH photonic bath, shown for a ring of 64 emitters alternating on A/B sublattices. The non-zero entries (*nz* = 480) reveal long-range, directionally biased couplings induced by the topological bath, while maintaining bipartite symmetry. (b) The corresponding energy spectrum exhibits a nearly symmetric distribution of eigenvalues for weak dimerization (*δ* = 0.05), indicative of emergent collective behaviour. This structure is consistent with an effective long-range XX model in the near-uniform coupling limit, where the bandgap closes and coherence is delocalized. (c) Ground-state spin-spin correlator $\sigma_{i}^{z}\sigma_{j}^{z}$ computed for six emitters, showing strong correlations within sublattices and alternating correlations across A/B pairs. The staggered structure reflects a double Néel-like ordering, characteristic of topological spin exchange mediated by the SSH bath. (d) Mutual information matrix between emitters, quantifying quantum correlations across the ring. High off-diagonal values, especially between distant A/B sites, highlight the presence of long-range entanglement induced by topological chiral interactions (*β* ^+^ directionality).

Two notable many-body phases appear in such a model, as revealed by exact diagonalization of finite size chains. For strong excitation bias $\left|\mu \right|$ , the system is fully polarized (all spins in $\left|g\right\rangle$ ). As $\left|\mu \right|$ is reduced, the system undergoes a phase transition and the spins can form an XY superfluid (for shorter-range interactions) or enter a long-range ordered phase depending on the range of $J_{mn}^{AB}$ . In particular, when Δ = 0 (in the middle of the topological gap), the system develops a double Néel order. This exotic configuration emerges from the alternating-sign, unidirectional couplings in Eq. (11) and has no analogue in conventional spin models. In an open chain, topological boundary modes would lead to uncoupled spins at the edges. However, in our ring geometry, these boundary spins are connected by periodicity. This lifts any degeneracy associated with edge modes and supports a uniformly extended double Néel state around the loop, a clear manifestation of topological coherence and symmetry breaking in many-emitter photonic systems. Figure 5a displays the sparsity pattern of the effective spin Hamiltonian *H*_spin_ with dimerization parameter *δ* = 0.5, showing that spin interactions are exclusively bipartite (*A* ↔ *B*) and exhibit long-range structure, a direct consequence of the chiral, directional Green’s function mediated by the SSH photonic ring.

In the special limit of *δ* → 0 (nearly uniform chain, *L*_BS_ → ∞), $J_{mn}^{AB}$ becomes effectively constant for all pairs. However, this uniform configuration leads to a high degree of symmetry in the emitter-mediated interactions but the photonic bandgap closes, eliminating any topological edge modes. Then Hamiltonian (11) simplifies via a unitary transformation *U* (flipping the sign of every other spin to remove the ${\left(-1\right)}^{j}$ factors to [40]:

(12) $$H_{\text{spin}}^{\prime }=UH_{\text{spin}}U^{t}\approx J\left(S_{A}^{+}S_{B}^{-}+S_{A}^{-}S_{B}^{+}\right)$$

where $S_{A}^{+}=\sum_{m}\sigma_{m,A}^{eg}$ and $S_{B}^{-}=\sum_{n}\sigma_{n,B}^{ge}$ represent collective raising/lowering operators for all emitters on sublattice A or B. This model captures coherent exchange between two large spins and supports a mean-field ferromagnetic order in the *XY* plane, with sublattices A and B anti-aligned in phase space. This ordered phase corresponds, in the original basis, to a double Néel-like configuration, but it is not topological. The corresponding energy spectrum (Figure 5b) reveals a narrow band of nearly symmetric eigenvalues (with dimerization parameter *δ* = 0.05), consistent with collective behaviour in a bipartite XX-type model, particularly in the near-uniform limit.

In contrast, for finite *δ* ≠ 0, the SSH ring exhibits a finite photonic bandgap and retains its topological band structure, enabling the emergence of topologically protected emitter modes. In this regime, the many-body physics intertwines with the geometry and topology of the lattice, leading to novel spin phases that are absent in trivial chains with *δ* = 0, means the existence of long-range coherence and robust spin phases in this system is intrinsically linked to the presence of a topological gap and its associated bound states. To probe the structure of the ground state, we evaluate equal-time spinspin correlations ${\langle \sigma }_{z}^{i}\sigma_{z}^{j}\rangle$ in Figure 5c. The resulting matrix shows staggered antiferromagnetic correlations on alternating sublattices, characteristic of the predicted double Néel order in chiral emitter-emitter interaction mediated by topological origin. The mutual information matrix (Figure 5d) further confirms the emergence of entanglement across nonlocal pairs, showing that the topological photonic environment supports long-range quantum correlations that are encoded into the effective spin model. These features would become more short-ranged or suppressed for higher dimerization (e.g., *δ* = 0.7), where the interactions become more localized and the many-body wavefunction flattens due to tighter confinement of the photonic bound states. The entanglement entropy plotted across bipartite cuts for different values of the dimerization parameter *δ* in Figure 4. Although the spatial range of the interaction becomes shorter in limit of $\left|\delta \right|\to 1$ , its coherence and robustness increase, leading to stronger entanglement and more structured mutual information matrices. This effect is particularly pronounced in the ring geometry, where periodic boundary conditions eliminate edge effects and allow topological modes to propagate unidirectionally without reflection. For intermediate-to-large *δ* (e.g., *δ* ≈ 0.5 − 0.7), we observe maximal entanglement entropy across emitter partitions and pronounced mutual information between distant pairs, highlighting the critical role of topological band structure in enabling robust many-body coherence.

Having established the theoretical underpinnings of topological emitter-bath dynamics in the SSH ring geometry, we turn to a realistic nanophotonic implementation capable of hosting such physics. We pattern a 40 nm gold film with 32 circular nanoholes (diameter *D* = 248 nm), arranged as 16 SSH unit cells in a closed ring. Alternating center-to-center spacings *S*_intra_ = 240 nm, *S*_inter_ = 260 nm (see Figure S_2_). A physics-informed deep-learning design targets a topological bandgap and strong mode confinement at *λ* ≈ 793 nm, consistent with prior plasmonic resonances observed near 789–793 nm. 20 Full-wave simulations (COMSOL) confirm an SSH-like plasmonic band structure with edge-localized modes (Figure S4) [25].

**Figure 6:**
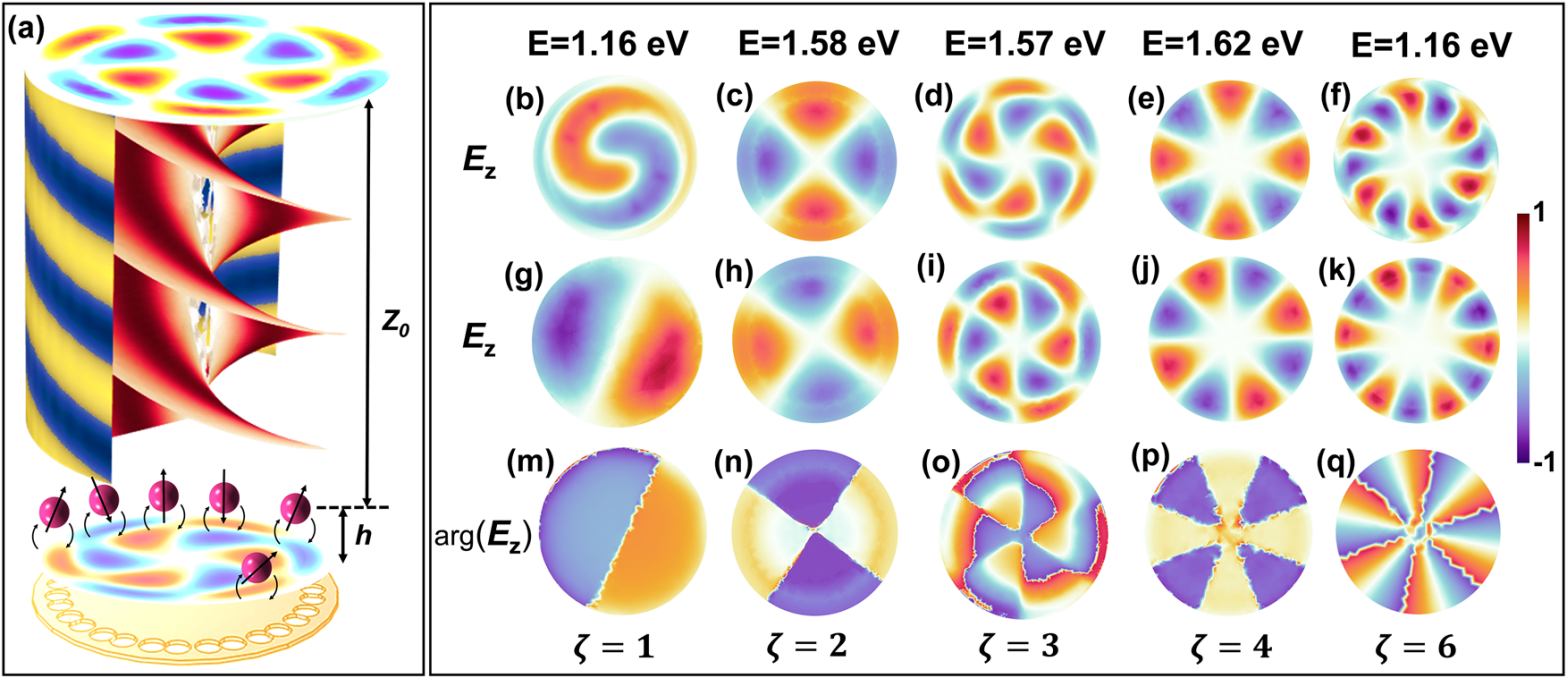
Chiral topological mode transfer from the SSH ring to the far field via emitter-enhanced vortex coupling. (a) Schematic illustration of the far-field propagation of topological optical modes in a nanophotonic SSH ring composed of 16 subwavelength holes patterned in a 40 nm-thick gold film. Gold nanospheres placed *h* = 200 nm above the ring act as localized dipolar emitters, coherently coupled to the vortex-like near fields supported by the SSH chain. The vertical propagation of these hybrid modes leads to far-field patterns carrying well-defined orbital angular momentum (OAM), enabled by spin–orbit coupling and enhanced through near-field overlap and Purcell enhancement. (b–f) Simulated spatial electric field component *E*_*z*_ in the near field above the ring for selected resonances at 1.16 eV, 1.58 eV, 1.57 eV, 1.62 eV, and 1.66 eV, respectively, showing vortex-like features characteristic of SSH topological band modes. (g–k) Corresponding far-field *E*_*z*_ field profiles recorded at a vertical height *Z*_0_ = 4 µm, revealing the radiation patterns imprinted by emitter-SSH mode coupling and collective coherence. (m–q) Phase distribution arg(***E***_*z*_) in the far field, showing quantized 2*πζ* phase winding around the beam center for winding numbers *ζ* = 1 to *ζ* = 5. The far-field topological charge equals the SSH mode winding number *ζ*; discrete azimuthal sampling by the *N* unit cells limits unique orders to $\left|\zeta \right|\leq N\;/2$ . The azimuthal phase singularities indicate the transfer of OAM from the topological modes of the SSH ring to the far field, enabled by coherent re-radiation from gold nanospheres acting as quantum emitters.

We place 60 nm gold nanospheres at selected holes as plasmonic quantum emitters. In the dipole limit, their optical response can be quantized as bosonic oscillators [41]:

(13) $${\hat{H}}_{\text{plasmon}}=\underset{\lambda }{\sum }\hbar \omega_{\text{plasmon}}\left({\hat{a}}_{\lambda }^{†}{\hat{a}}_{\lambda }+\frac{1}{2}\right)$$

where ${\hat{a}}_{\lambda }$ , ${{\hat{a}}^{†}}_{\lambda }$ are annihilation/creation operators and *λ* = 1 for dipole, selectively coupling to vortex-like edge modes with OAM and spin–orbit locking (see Figure 6b–f). The topological modes in SSH ring, exhibit vortex-like electric fields, described by a twisted phase structure $E_{\text{SSH}}\left(\rho ,\varphi \right)\approx E_{0}{\text{e}}^{j\left(\zeta \varphi +k_{r}\rho \right)}$ , and *k*_*r*_ is the radial wavevector [42] (see Supplementary Information Sections IV and V). which induce a dipole **P** ∝ **E**, with angular-momentum index *ζ* enabling spin-momentum locking (Supplementary Section IV) [43]. Only one circular dipole component (e.g. *σ* ^+^) couples efficiently to a given propagation direction, producing chiral excitation. The coupling is captured by the local field overlap and the Purcell factor [44]:

(14) $$\begin{array}{ll} F_{P} & =\frac{\Gamma_{\text{SSH}}}{\Gamma_{0}}=\frac{\text{Im}\left[P^{*}\cdot E_{\text{SSH}}\right]}{\text{Im}\left[P^{*}\cdot E_{\text{Vac}}\right]}\\ & =\frac{6\pi c^{3}}{\omega^{3}}d_{\text{eff}}^{t}\cdot \text{Im}\;G\left(r_{0},r_{0},\omega \right)\cdot d_{\text{eff}} \end{array}$$

which is directional and spin-selective in our topological bath, $d_{\text{eff}}\propto \sqrt{\hbar \omega \text{Re}\left[\alpha \left(\omega \right)\right]}$ [45] is the effective dipole moment. The photonic Green tensor $G\left(r_{0},\omega \right)$ governs both collective decay Γ_*mn*_ and coherent Lamb shifts *J*_*mn*_ in Eq. (3). The resulting interactions in the gap are long-ranged and chiral, with range set by *L*_BS_. In the gap regime, Γ → 0 and coherent exchange dominates, enabling many-emitter entanglement and long-range spin coherence (Supplementary Section V).

Plasmonic emitters seeded by the topological mode re-radiate coherently to the far field with a vortex phase profile (Figure 6a). The radiated field [46]:

(15) $$E_{\text{far}}\left(r,t\right)∼\overset{N}{\underset{j=1}{\sum }}\alpha_{j}\left(t\right)G_{\text{far}}\left(r,r_{j}\right)\cdot d_{\text{eff,j}}$$

$\alpha_{j}\left(t\right)=\langle {\hat{a}}_{j}\left(t\right)\rangle$ is the collective coherent amplitude of each emitter *j*, inherited from the spin ground state. The sum combines the emitter contributions coherently; helicity $\left(\sigma^{\pm }\right)$ , emitter placement, and the dimerization *δ* set the propagation direction (the chirality) and OAM sign, while the gap-set localization length controls the coupling range, reconfigurable directionality and cascaded many-emitter dynamics robust to disorder. This topological mechanism yields functionalities, isolation-like transport without magneto-optics, sublattice-addressable routing, and coherence-preserving exchange, that are not available in the similar molecules.

Simulations (Figure 6b–q) show clear 2*πζ* phase windings, i.e., far-field OAM equal to the SSH winding number *ζ*, linking near-field bound-state physics to far-field observables and providing an experimental signature of chiral coupling and of the underlying topological mode. In a ring with *N* unit cells (here *N* = 16), discrete SSH winding number $\left|\zeta \right|\leq N\;/2$ (here *N* = 16). In practice, the coupling efficiency decreases for very high $\left|\zeta \right|$ , so the cleanest emission appears for lower $\left|\zeta \right|$ [47].

## 7 Conclusions

Our results establish a flexible route to coherence-preserving, long-range emitter networks using topological plasmonic baths. This approach is not limited to the SSH chain; the same emitter–bath strategy can be extended to other topological lattices beyond the SSH ring. Chiral bound states at mid-gap generate unidirectional, exponentially ranged couplings whose signs and sublattice structure are set by the SSH topology which are not addressed in earlier SSH ring demonstrations [48]. These couplings drive non-Markovian revivals, robust coherence, and long-range entanglement that we capture with an effective bipartite spin model featuring a double Néel phase. The ring geometry is crucial: periodicity eliminates edge reflections, protects directionality, and supports uniform many-body order. Beyond our specific implementation, the principles, topological guidance, chirality, and collective spin coherence extend naturally to hybrid platforms including exciton–polaritons [49], [50], magnons [51], plexcitons [49], [52], and layered 2D materials [43], [49], [53], [54], [55], [56]. The same Green-tensor-encoded interactions can be engineered in dielectric metasurfaces and photonic crystals, enabling spin-selective quantum interfaces that are robust to disorder and operate beyond conventional decoherence limits. Whereas other similar photonic structures exhibit OAM and spin–orbit effects, our SSH ring realizes gap-protected, sublattice-chiral bound states that mediate unidirectional, exponentially ranged emitter couplings. Combining our architecture with active tuning (electro-optic or thermo-optic control of *δ* and *ω*_*e*_) would permit *in-situ* reconfiguration of interaction range, chirality, and many-body phase, while integrating single-photon detectors or on-chip gratings would facilitate direct readout of vortex-beam emission and correlation functions.

## 8 Methods

### 8.1 Analytical calculations

To explore the quantum optics and mutual coherence, I used home-built code. Full details about the parameters and simulation times are presented in Supplementary Notes.

### 8.2 Surrogate active physics-informed deep learning

For the design of SSH ring we used active physics-informed deep learning [25]. In this work, we developed a physics-informed deep learning framework that embeds key analytical constraints into the learning process and uses fast surrogate models to explore design space efficiently.

Model overview: The network is trained to map a compact set of geometric and coupling parameters. (nanohole diameter, intra/inter-dimer gap, dimerization, and number of unit cells) and excitation wavelength to three physically meaningful targets: coupling regime, topological character, and propagation direction. Physical knowledge enters the model in two ways: (1) feature engineering and labels derived from analytical scaling (e.g., plasmon-ruler/near-field coupling trends) and tight-binding theory; and (2) loss terms that penalize violations of expected behaviours (e.g., bandgap opening with dimerization, sublattice/chirality consistency, and monotonic dependence of localization length on $\left|\nu /w\right|$ . By incorporating these constraints, such as plasmonic mode coupling laws, the network can rapidly approximate topological properties from inputs like hole size, lattice spacing, and coupling strength without exhaustively solving Maxwell’s equations. Guided by the flowchart in Figure S5, we trained the model in three sequential tasks using a compact dataset of ∼4,000 simplified simulations. The optimized ring of perforated nanoholes designed with our physics informed deep learning exhibits a topological edge mode that propagates unidirectionally under non-planar excitation, with experimental operating conditions encoded during training.

### 8.3 Numerical simulations

We numerically corroborated the quantum-optical analysis with full-wave finite-element simulations (COMSOL Multiphysics, Wave Optics Module). Candidate geometries were first identified by our physics-informed surrogate model, then verified by solving the frequency-domain Maxwell equations using the electromagnetic waves, frequency domain interface. The device consists of a dimerized SSH ring perforated in a 40-nm gold film; 60-nm gold nanospheres were positioned at selected nanoholes and modelled as dipolar plasmonic emitters. We evaluated non-planar (electron-beam-like) and optical excitations, and we obtained near-field distributions (|E| and phase) together with far-field radiation patterns. These simulations confirm robust one-way edge transport and vortex-like emission in the optimized designs.

## Supplementary Material

Supplementary Material Details

Supplementary Material Details

Supplementary Material Details

Supplementary Material Details

Supplementary Material Details

Supplementary Material Details
