# Supplementary material for: From bound states to quantum spin models: chiral coherent dynamics in topological photonic rings
Source: Nanophotonics. 2025 Nov 25;14(24):4397–409. doi: 10.1515/nanoph-2025-0473 (PMC12704495; doi:10.1515/nanoph-2025-0473)
Supplement: Supplementary file 1 — Supplementary Material Details [file j_nanoph-2025-0473_suppl_001.pdf]

Supporting Information

From Bound States to Quantum Spin Models: Chiral Coherent Dynamics in Topological Photonic Rings

Fatemeh Davoodi[1,2,*]

[1]*Nanoscale Magnetic Materials, Institute of Materials Science, Kiel University, 24143, Kiel, Germany*

[2]*Kiel Nano, Surface and Interface Science KiNSIS, Christian Albrechts University, Kiel, Germany*

[*]*fda@tf.uni-kiel.de*

In this Supporting Information, we provide further details on tight binding Hamiltonian in SSH plasmonic chain, linear and ring shape, chiral emitter–bath interaction in the thermodynamic limit, single-emitter bound states and non-Markovian dynamics, light–matter interaction: from quantized dipoles to effective spin coupling, Purcell-enhanced coupling and far-field transfer by gold nanosphere:

I. Tight Binding Hamiltonian in SSH Plasmonic Chain, Linear and Ring Shape
II. Chiral Emitter–Bath Interaction in the Thermodynamic Limit
III. Single-Emitter Bound States and Non-Markovian Dynamics
IV. Light–Matter Interaction: From Quantized Dipoles to Effective Spin Coupling
V. Purcell-Enhanced Coupling and Far-Field Transfer by Gold Nanosphere
VI. Flowchart of the training and searching process for the proposed algorithm

Section I: Tight Binding Hamiltonian in SSH Structures

I.1 Tight Binding Hamiltonian in SSH Plasmonic Chain

To model the plasmonic Su–Schrieffer–Heeger (SSH) chain, we employ the tight-binding (TB) approximation, which captures nearest-neighbor hopping between localized plasmonic modes in nanoparticles arranged in a dimerized lattice. The TB Hamiltonian for a linear SSH chain is written as [1,2]

$$\hat{H} = v\sum_{m=1}^{n}(|m,B\rangle\langle m,A| + h.c.) + w\sum_{m=1}^{n-1}(|m+1,A\rangle\langle m,B| + h.c.) \quad (1)$$

where:

$|m,\alpha\rangle = |m\rangle \otimes |\alpha\rangle, m = 1,\dots,N, \alpha = A,B$

v and w denote the intra-cell and inter-cell coupling constants, respectively.

This can also be written in a matrix form using Pauli operators acting on sublattices:

$$\hat{H} = v\sum_{m=1}^{n}(|m,B\rangle\langle m| \otimes \sigma_x + h.c.) + v\sum_{m=1}^{n-1}\left(|m+1\rangle\langle m| \otimes \frac{\sigma_x + i\sigma_y}{2} + h.c.\right) \quad (2)$$

To analyze the band structure, we apply a Bloch ansatz for bulk eigenstates:

$$|k\rangle = \frac{1}{\sqrt{N}}\sum_{m=1}^{N} e^{imk}|m\rangle, k \in \left\{\frac{2\pi}{N}, \frac{4\pi}{N}, \ldots, \frac{2N\pi}{N}\right\}$$

The full eigenstate is $|\psi_n(k)\rangle = |k\rangle \otimes |u_n(k)\rangle$, with:

$|u_n(k)\rangle = a_n(k)|A\rangle + b_n(k)|B\rangle, n = 1,2$

The momentum-space Hamiltonian becomes[3]

$$H(k) = \begin{pmatrix} 0 & v + we^{ik} \\ v + we^{ik} & 0 \end{pmatrix} \Rightarrow E_{\pm}(k) = \pm|v + we^{ik}| = \pm\sqrt{v^2 + w^2 + 2vw\cos(k)} \quad (3)$$

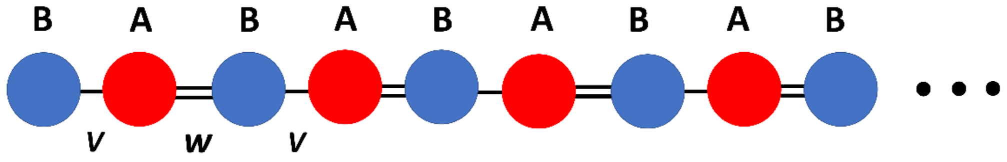

Figure S1: Linear SSH chain with dimerized hopping amplitudes. Schematic of a one-dimensional Su–Schrieffer–Heeger (SSH) chain consisting of alternating A (red) and B (blue) sublattice sites. The chain exhibits dimerized nearest-neighbor hopping characterized by intra-cell coupling v and inter-cell coupling w, leading to a two-site unit cell. The alternation of strong and weak bonds defines the dimerization parameter $\delta=(v-w)/(v+w)$, which determines the topological phase of the chain. When $v<w$, the chain is in the nontrivial topological phase supporting edge states under open boundary conditions.

The off-diagonal term encodes the alternating hopping:

$$f(k) = -J[(1+\delta) + (1-\delta)e^{-ik}] \quad (4)$$

where $v = J(1+\delta)$ and where $w = J(1-\delta)$ are the intra-dimer and inter-dimer coupling amplitudes, respectively. (Here $0 < |\delta| < 1$ is the dimerization parameter).

Diagonalizing Equation (1) yields two photonic bands (upper and lower) given by the dispersion $\omega_{\pm}(k) = \omega_a \pm \omega(k)$. Here $\omega(k) = |f(k)|$ is the positive band energy, found to be:

$$\omega(k) = J\sqrt{2(1+\delta^2) + 2(1-\delta^2)\cos k} \quad (5)$$

In the continuum limit ($N \to \infty$), k is continuous on $[-\pi, \pi]$. For a finite ring of N unit cells, k takes quantized values $k = \frac{2\pi m}{N}$ ($m = 0,1, \ldots N-1$), but still satisfies Equation (3). The two bands are symmetric about ω_a (a consequence of sublattice chiral symmetry). They span frequencies $\omega \in [-2J, -2|\delta|J]$ (lower band) and $\omega \in [2|\delta|J,\ 2J]$ (upper band), leaving a bandgap of width $4|\delta|J$ around zero (which closes when ($\delta \to 0$), recovering a uniform chain) (See Fig.1 (b,d) in the main text.)

I.2.Tight Binding Hamiltonian in ring

To simulate a finite SSH ring, we introduce periodic boundary conditions and model the system as a tight-binding loop with complex phase hopping.

The Hamiltonian becomes [4]

$$H = \sum_n v\left(e^{i\theta}a_{\alpha,n}^{\dagger}a_{\beta,n} + e^{-i\theta}a_{\beta,n}^{\dagger}a_{\alpha,n}\right) + \sum_n w\left(e^{i\theta}a_{\beta,n}^{\dagger}a_{\alpha,n+1} + e^{-i\theta}a_{\alpha,n+1}^{\dagger}a_{\beta,n}\right) \quad (6)$$

with $a^{\dagger}_{\alpha,n}$, $a_{\alpha,n}$ are bosonic creation and annihilation operators on sublattice $\alpha \in \{A, B\}$ in unit cell n, $\theta = {}^{2\pi}/_{N}$ is a Peierls phase encoding the ring geometry, N is the total number of sites in the ring, the site number with $N + 1 = 1$.

In this formulation, δ (the dimerization parameter) is encoded geometrically via the angular spacing and coupling variation in the ring. A nonzero $\delta = {}^{(S_{inter} - S_{intra})}/_{S_{inter} + S_{intra}}$ corresponds to asymmetry in the hopping parameters v and v.

The superlattice geometry (periodic modulation of coupling) or interference between multiple domain walls and the band eigenvalues on the Bloch circle (from diagonalizing $H_B(k)$ in Equation (6)) show the Bloch vector $d(k) = (\mathrm{Re}[f(k)], \mathrm{Im}[f(k)])$ wrapping around the origin more than once (Fig. S3), leads to have higher winding number and more edge states [4].

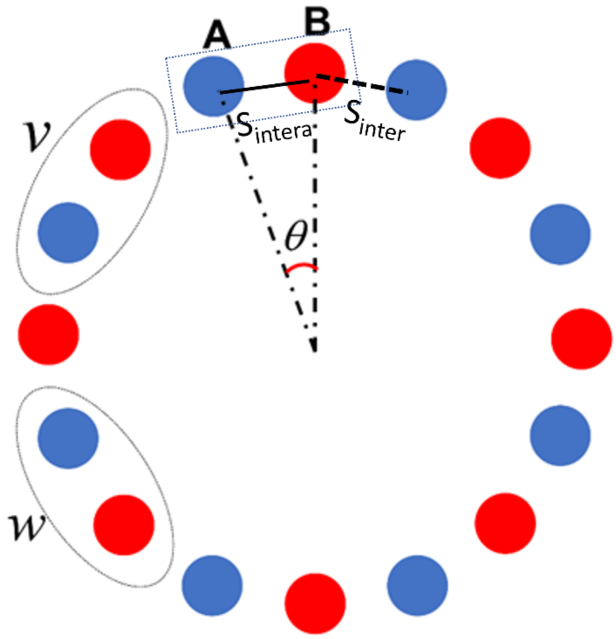

Figure S2: Schematically illustrates the SSH ring structure composed of 2N=16 sites. Each unit cell consists of a red (A) and blue (B) nanodisk, with alternating intra-cell and inter-cell spacings. The angular separation between adjacent nanodisks defines the effective dimerization δ and topological phase of the chain.

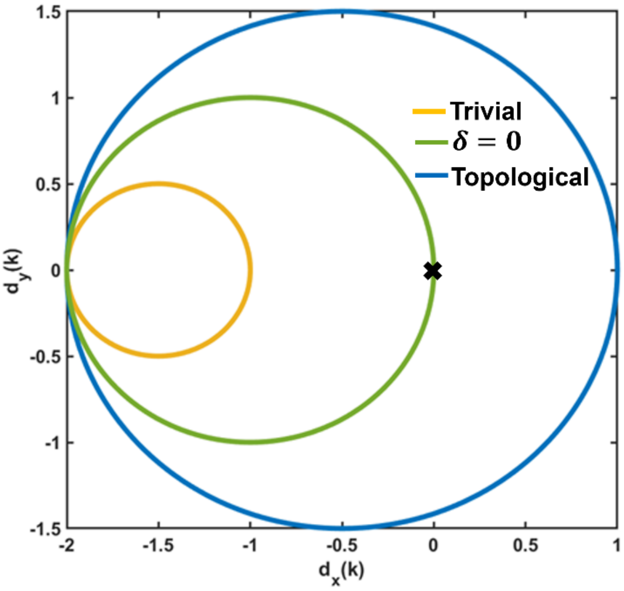

Figure S3: Winding behavior of the SSH model in $\boldsymbol{d(k)}$-space for different dimerization regimes. The parametric curves represent the trajectory of the Bloch Hamiltonian vector $d(k) = \big(d_x(k), d_y(k)\big)$ across the Brillouin zone for three cases: topological (blue), critical ($\delta = 0$, green), and trivial (yellow). The number of times each curve encircles the origin determines the winding number ζ, a topological invariant characterizing the SSH phase. Only the topological case (blue) encloses the origin, yielding $\zeta \geq 1$, while the trivial case (yellow) does not, giving $\zeta = 0$. The gapless transition point (green) lies exactly tangent to the origin. The black '×' marks the origin in $d(k)$-space. This plot visualizes how the SSH model's topological classification arises from phase winding in momentum space.

Section II: Chiral Emitter–Bath Interaction in the Thermodynamic Limit

In this section, we analytically derive the effective emitter-emitter interaction mediated by the SSH bath in the thermodynamic limit $N\rightarrow\infty$, and explain how chiral and nonreciprocal behavior emerges from topological band structure and the Lindblad master equation.

II.1 Non-Hermitian Dynamics and Chirality

As observed in Fig. 2 of the main text, the excitation of emitter 1 strongly couples to topological edge modes, while emitter 2 shows a delayed and weaker response. This asymmetry results from the unidirectional flow of energy allowed by the chiral SSH bath, where the emitter-bath interaction is described by a non-Hermitian master equation [5]:

$$\rho = -\imath[\dot{H}_S + H_{LS}, \rho] + \sum_{m,n} \frac{\Gamma_{mn}^{ab}}{2}\left(2\sigma_n^{ge}\rho\sigma_m^{eg} - \sigma_m^{eg}\sigma_n^{ge}\rho - \rho\sigma_m^{eg}\sigma_n^{ge}\right) \tag{7}$$

This cascade-type Lindblad form predicts nonreciprocal energy exchange, where emitter 1 can influence emitter 2, but not vice versa, manifesting as the chiral β^+ interaction (see Fig. 2e-f).

II.2 Evaluating Emitter–Emitter Interaction Kernels

To gain analytical insight into this behavior, we consider the thermodynamic limit of the SSH Hamiltonian [6]:

$$H_B = \sum_k \left[a_k^\dagger \; b_k^\dagger\right] \begin{bmatrix} \omega_a & f(k) \\ f^*(k) & \omega_a \end{bmatrix} \begin{bmatrix} a_k \\ b_k \end{bmatrix}, \text{ with } f(k) = -J\left[(1+\delta) + (1-\delta)e^{-ik}\right] \tag{8}$$

We analytically compute the emitter–emitter interaction Green's function $\sum_{mn}^{ab}(\omega)$ by converting the real-space lattice sum to a momentum integral.

II.3 Same-Sublattice (AA or BB) Coupling

For two emitters on the same sublattice a=b, we obtain [7,8]:

$$\Sigma_{mn}^{AA}(\omega) = -\frac{g^2\omega}{2}\left[y_+^{|x_{mn}|} + Q_+(y_+) - y_-^{|x_{mn}|}Q_-(y_-)\right]\frac{1}{\sqrt{\omega^4-4J^2(1+\delta^2)\omega^2+16J^4\delta^2}} \quad (9)$$

SII.4 Cross-Sublattice (AB or BA) Coupling

For emitters on opposite sublattices:

$$\Sigma_{mn}^{AA}(\omega) = -\frac{g^2J}{2}\left[F_{x_{mn}}(y_+)Q_+(y_+) - F_{x_{mn}}(y_-)Q_-(y_-)\right]\frac{1}{\sqrt{\omega^4-4J^2(1+\delta^2)\omega^2+16J^4\delta^2}} \quad (10)$$

Here, $F_n(z) = (1+\delta)z^{|n|} + (1-\delta)z^{|n+1|}$, $Q_\pm$ are Heaviside functions that enforce energy conservation within the light cone.

II.5 Role of $y_\pm$: Localized vs Radiative Modes

The complex parameters $y_\pm$ encode the exponential decay of bound states:

$$y_\pm = \frac{\omega^2-2J^2(1+\delta^2)\pm\sqrt{\omega^4-4J^2(1+\delta^2)\omega^2+16J^4\delta^2}}{2J^2(1-\delta^2)} \quad (11)$$

Whether $|y_\pm| < 1$ or > 1 determines if a bound state is localized (inside bandgap) or delocalized (in band). When ω lies in the bandgap, $\mathrm{Im}[\Sigma\ ] = 0$, and only coherent exchange persists. Otherwise, dissipative decay dominates.

II.6 Interpretation for Finite Ring

For a finite SSH ring, the momentum becomes discrete: $k = \frac{2\pi m}{N}, \quad m = 0, \dots N-1$

The integrals over k are replaced by discrete sums, and step $Q_\pm$ become Kronecker deltas. However, the essential physics remains: chirality and nonreciprocal exchange arise from the complex phase of the eigenmodes, and sharp features in $\Sigma(\omega)$ near the band edges indicate the emergence of topological bound states.

The asymmetry in coupling seen in Fig. 2d (main text) arises from the imaginary part of Σ_{mn}^{ab} , while the energy shifts (Lamb shifts) originate from its real part**:**

- Coherent coupling: $J_{mn}^{ab} = \mathrm{Re}\,\Sigma_{mn}^{ab}$
- Dissipative rate: $\Gamma_{mn}^{ab} = 2\mathrm{Im}\,\Sigma_{mn}^{ab}$

This establishes a microscopic origin for chiral Lindblad dynamics in Eq. (8) of the main text.

Section III: Single-Emitter Bound States and Non-Markovian Dynamics

When a single quantum emitter (QE) is coupled to the SSH ring and its transition frequency lies inside a photonic bandgap, spontaneous emission into propagating modes is forbidden. Instead, the system supports a localized bound state (BS): a hybrid light-matter excitation where the photon field is exponentially localized around the emitter. This phenomenon arises from the absence of decay channels, and is a hallmark of non-Markovian and topological physics in structured baths.

III.1. Single-Excitation Ansatz

To study this phenomenon, we restrict the Hilbert space to the single-excitation sector, where the total system contains one quantum of excitation (either in the emitter or in the bath). We use the ansatz:

$$|\Psi(t)\rangle = C_e(t)|e;\mathrm{Vac}\rangle + \sum_{j,\alpha\in\{A,B\}} C_{j,\alpha}(t)|g;1_{j,\alpha}\rangle \quad (12)$$

where:

$|e;\mathrm{Vac}\rangle$: emitter is excited, bath is in vacuum,

$|g;1_{j,\alpha}\rangle$: emitter is in the ground state and a single photon is at site (j,α)

$C_e(t)$: amplitude of emitter being excited;

$C_{j,e}(t)$: amplitude for a photon at lattice site (j, α)

III.2. Self-Energy and the Bound-State Equation

Inserting this ansatz into the Schrödinger equation with the full Hamiltonian $H = H_B + H_{int} + H_e$, and going to the Laplace domain yields an effective energy-dependent self-consistency equation:

$$E_{BS} = \Delta + \sum_{ee}(E_{BS}) \tag{13}$$

Where Δ is the emitter detuning from the band center, and $\sum_{ee}(E_{BS}) = \sum_{nn}^{AA}(E_{BS})$ is on-site self energy, capturing the back action of the bath on the emitter. The emitter's Green's function is defined as:

$$G_e(z) = \frac{1}{z-\Delta-\sum_{ee}(z)} \tag{14}$$

This equation admits real-valued solutions E_{BS} only inside bandgaps, where $\mathrm{Im}\sum = 0$ indicating no dissipation and the existence of a bound state.

In practice, Equation (12) can have up to three solutions for E_{BS} ; one in each bandgap (lower, middle, upper); because $\sum_{ee}(Z)$ diverges at all band edges.

III.3. Explicit Wavefunction of the Bound State

Assuming the emitter is coupled to site *j*=0, sublattice A, the wavefunction components are (as shown in equation 14 in the main text) [5,7]

$$C_{j,A} = \frac{gE_{BS}C_e}{2\pi}\int_{-\pi}^{\pi}\frac{e^{ikj}}{E_{BS}^2-\omega^2(k)}dk, \tag{15}$$

$$C_{j,B} = \frac{gC_e}{2\pi}\int_{-\pi}^{\pi}\frac{\omega(k)e^{i[kj-\phi(k)]}}{E_{BS}^2-\omega^2(k)}dk \tag{16}$$

where $f(k) = \omega(k)e^{i\phi(k)}$ is the Bloch off-diagonal coupling function, $\omega(k)$ is the dispersion relation, $\phi(k)$ is the phase winding angle related to topology (see also the winding number discussion in Section SI). The photonic component of the bound state is spatially localized, with decay length set by the inverse of the imaginary part of the complex momentum, which becomes large near the band edges.

III.4. Normalization and Physical Interpretation

The wavefunction is normalized such that:

$$|C_e|^2 + \sum_{j,\alpha}|C_{j,\alpha}|^2 = 1 \tag{17}$$

The emitter excitation probability at long times (steady-state) is:

$$P_e(\infty) = |C_e|^2 = \text{nonzero} \tag{18}$$

This is a direct consequence of the system forming a dark bound state, the emitter cannot fully decay because no bath states are resonantly accessible. For example, at detuning Δ=0 (center of the middle bandgap), one finds:

$$|C_e|^2 = \left[1 + \frac{g^2}{4J^2|\delta|}\right]^{-2} \tag{19}$$

showing that decreasing δ (closing the gap) reduces the excitation retention. As $\delta \to 0$, the bandgap closes, and the emitter decays fully returning to the Markovian limit. The non-exponential, oscillatory behavior observed in time-domain simulations when $|\delta| \to 1$ (Fig. 3 in the main text), stems from the non-Markovian memory kernel of the SSH bath.

III.5. Chiral localization

A striking feature of the SSH bath is that a mid-gap bound state ($|\Delta| \sim 0$) localizes asymmetrically to one side of the emitter, a chiral localization determined by the topology (sign of δ). In the ring, this means

the bound photonic excitation propagates predominantly in one direction around the loop. To see this, consider the analytic solution at $\Delta = 0$ (exact mid-gap) for an emitter on sublattice A at cell 0. In the trivial phase, the bound state has energy $E_{BS} = 0$ and the wave amplitudes evaluate to:

$$C_{j,A} = 0, \quad C_{j,B} = gC_e(-1)^j \frac{1}{J(1+\delta)} \left(\frac{1-\delta}{1+\delta}\right)^j \; for \; j \geq 0 \tag{20}$$

and $C_{j,B} = 0$ for $j < 0$. This solution is nonzero only on the B sublattice and only for lattice sites in front of the QE (here $j \geq 0$ labels the cells in the clockwise direction, assuming we number cells such that $j = 0$ at the emitter). In topologically nontrivial phase, the roles are reversed: the bound state populates only A-sublattice sites on one side of the emitter (and decays in the opposite direction). In either case, the localization length is $L_{BS} = -\frac{1}{ln\left|\frac{1-\delta}{1+\delta}\right|}$. For small $L_{BS} \approx \frac{1}{2|\delta|}$ for $|\delta| \ll 1$, which can become comparable to the circumference of the ring. If L_{BS} approaches N (when the gap nearly closes), the bound state's evanescent tail can wrap around the ring and meet the "back" of the emitter. In that regime the strictly one-sided solution (18) is not unique, the clockwise and counter clockwise decaying solutions hybridize into two standing-wave modes (one symmetric, one antisymmetric). However, for a moderate ring size (for example, 16 cells) and a reasonably sized gap, the bound state is effectively chiral and confined to one side of the QE. This can be intuitively understood as the emitter acting as a topological boundary in the middle of the ring, with the bound state resembling an "edge state" localized adjacent to that boundary.

Section IV: Light–Matter Interaction: From Quantized Dipoles to Effective Spin Coupling

IV.1. Quantized Plasmonic Dipole of Gold Nanospheres

In our system, 60 nm gold nanospheres are positioned near selected nanoholes in a patterned plasmonic SSH ring. Though these are classically described particles, when subwavelength and operating near resonance, their dominant dipolar plasmonic modes can be modeled quantum mechanically. Each nanosphere supports localized surface plasmon (LSP) resonances, and their quantized response is captured by a bosonic Hamiltonian:

$$\hat{H}_{plasmon} = \sum_\lambda \hbar\omega_\lambda \left(\hat{a}_\lambda^\dagger \hat{a}_\lambda + \frac{1}{2}\right) \tag{21}$$

where $\hat{a}_\lambda$, $\hat{a}^\dagger{}_\lambda$ satisfying $[\hat{a}_\lambda, \hat{a}_{\hat{\lambda}}^\dagger] = \delta_{\lambda\hat{\lambda}}$ are annihilation and creation operators of the plasmon mode with resonance frequency ω_λ. The Hamiltonian is derived from the classical energy $H = \int d^3r(\varepsilon_0 \mathbf{E}_\perp^2 + \mu_0^{-1}\mathbf{B}^2)$ with considering transverse electric and magnetic field operators [9]

$$\mathbf{E}_\perp(\mathbf{r}) = i\sum_{\mathbf{k},\lambda} \sqrt{\frac{\hbar\omega_k}{2\varepsilon_0 V}} [a_{\mathbf{k}\lambda}\mathbf{e}_{\mathbf{k}\lambda}e^{i\mathbf{k}.\mathbf{r}} - \text{h.c.}] \tag{22}$$

$$\mathbf{B}(\mathbf{r}) = i\sum_{\mathbf{k},\lambda} \sqrt{\frac{\hbar}{2\varepsilon_0\omega_k V}} [a_{\mathbf{k}\lambda}(\mathbf{K} \times \mathbf{e}_{\mathbf{k}\lambda})e^{i\mathbf{k}.\mathbf{r}} - \text{h.c.}] \tag{23}$$

For nanospheres much smaller than the wavelength (diameter $\ll$ wavelength), the dipole mode (λ=1 for dipole) dominates the optical response.

The quantized dipole operator in the time domain becomes:

$$\hat{\mathbf{P}}(t) = d_{eff}(\hat{a}e^{-i\omega t} + \hat{a}^\dagger e^{i\omega t}) \tag{24}$$

where $\hat{a}$, $\hat{a}^\dagger$ are bosonic operators, and d_{eff} is effective dipole moment vector and is related to the polarizability $\alpha(\omega)$ of the particle:

$$d_{eff} \propto \sqrt{\hbar\omega \mathrm{Re}[\alpha(\omega)]} \tag{25}$$

When the excitation is driven by helicity-resolved structured fields (e.g. the topological mode from the SSH ring), the plasmon couples preferentially to either $\hat{\sigma}^{+}$ or $\hat{\sigma}^{-}$ polarizations. This spin selection makes the plasmon act like a circular dipole with transition operators: $\hat{\sigma}^{+} \sim \hat{a}$, $\hat{\sigma}^{-} \sim \hat{a}^{\dagger}$.

Thus, each nanosphere can act like an effective two-level system, with:

- Ground state: no excitation $|0\rangle$
- Excited state: one plasmon excitation $|1\rangle$

When exposed to a structured photonic environment (e.g., an SSH ring), the nanosphere interacts with the local electric field $\mathbf{E}_{loc}(r_0, t)$ and the interaction Hamiltonian reads:

$$\hat{H}_{int} = -\hat{P} \cdot \mathbf{E}_{loc}(r_0, t) \tag{26}$$

which describes the exchange of energy between the nanosphere's plasmonic mode and the structured field. In the rotating-wave approximation (RWA), this can be recast in terms of emitter–mode coupling as [10,11]

$$H_{int} = \sum_{m,v} \hbar g_v(r_m)\, \sigma_m^{+} a_v + \hbar g_v^{*}(r_m) \sigma_m^{-} a_v^{\dagger} \tag{27}$$

m is index of the emitter, $g_v(r_m) = -d_m \cdot E_v(r_m)/\hbar$ represents the complex coupling strength between emitter m and v, $\sigma_m^{\pm}$, a_v, $a_v^{\dagger}$ are the raising/lowering operators for the emitter and annihilation/creation operators for the bath modes, respectively.

The bath refers to the continuum of plasmonic modes, like all the ways light or plasmons (quasi-particles of electron density waves) can move in the system. Which can be expressed by:

$$H_{bath} = \sum_v \hbar\omega_v a_v^{\dagger} a_v \tag{28}$$

IV.2. Mode-Resolved Excitation Spectrum of a Chiral Dipole in a Finite SSH Ring

The topological modes in SSH ring, exhibit vortex-like electric fields (shown in Figure S4), described by a twisted phase structure $E_{SSH}(\rho, \varphi) \approx E_0 e^{j(\zeta\varphi + k_r\rho)} \hat{e}_{\pm}$, and k_r is the radial wavevector. These modes carry orbital angular momentum (OAM), $e^{j(\zeta\varphi)}$ and the spin angular momentum (polarization) $\hat{e}_{\pm}$, enabling spin-orbit coupling at the nanoscale. ζ, determines the quantized angular momentum and act as the winding number in the SSH ring, because the phase advances by $2\pi\zeta$ when the azimuthal angle φ goes once around the ring. This comes from the periodic boundary condition in the SSH ring chain:

$$E\big(\varphi(k) + \delta\varphi(k)\big) = exp\big(i\zeta\delta\varphi(k)\big)E\big(\varphi(k)\big)\,,\ \delta\varphi(k) = \frac{2\pi}{N} \text{ and } \zeta = 0, 1, \ldots, N\text{-}1 \tag{29}$$

so that the field picks up a fixed phase increment at each of the N sites.

Each quantized angular momentum state ζ can be decomposed into two helical components $m = \pm\zeta$ corresponding to clockwise or counter-clockwise rotation. A σ^{+} polarized excitation couples preferentially to the $m = +\zeta$ component (right-handed rotation). In this way, the OAM is directly linked to the topological SSH mode: it is a property of the imposed azimuthal phase factor $e^{j(\zeta\varphi)}$, which shapes the wavefront and fixes the topological charge ζ. The OAM is therefore quantized, and different values of ζ label distinct optical modes. When the electric field in Eq. (29) undergoes ζ phase windings, the associated OAM also winds ζ times around the z-axis (Figure S4). Because of the circular symmetry of the ring, both positive and negative ζ are allowed, leading to the observed directional (chiral) optical behaviour. Figure S5 confirms that, while the longitudinal component Ez shows a $2|\zeta|$-lobe pattern due

to projection and discrete sampling, the polarization-summed intensity remains annular and the OAM spectrum is dominated by the target order $m=\zeta$.

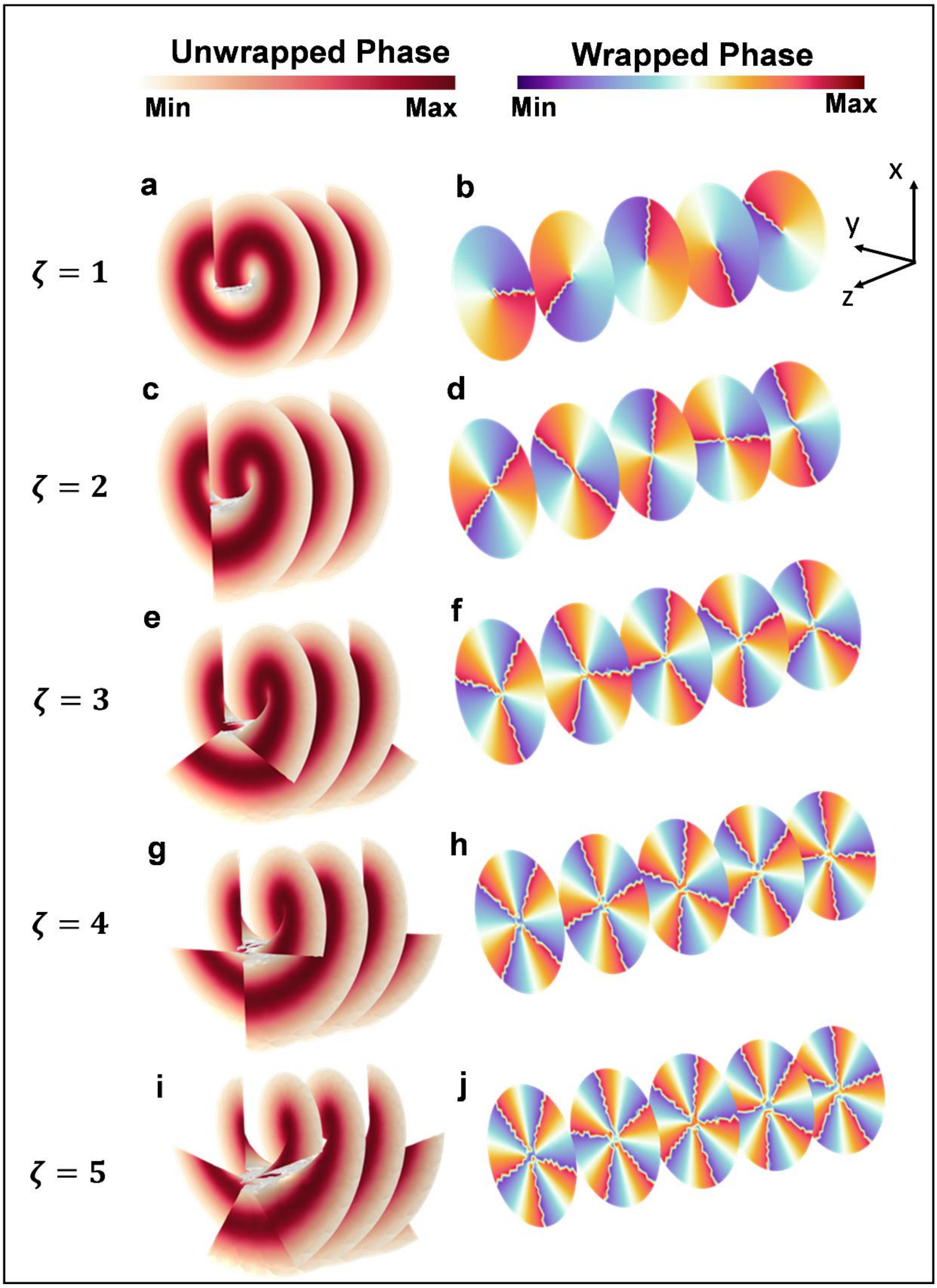

Figure S4: Unwrapped (left) and wrapped (right) far-field phase distributions for SSH-ring modes with increasing winding number ζ. Panels a, c, e, g, and i show the unwrapped phase, where the phase around the beam center increases smoothly by $2\pi\zeta$, confirming the helical nature of the field. Panels b, d, f, h, and j show the corresponding wrapped phase; the apparent $\pm\pi$ jumps arise from phase wrapping.

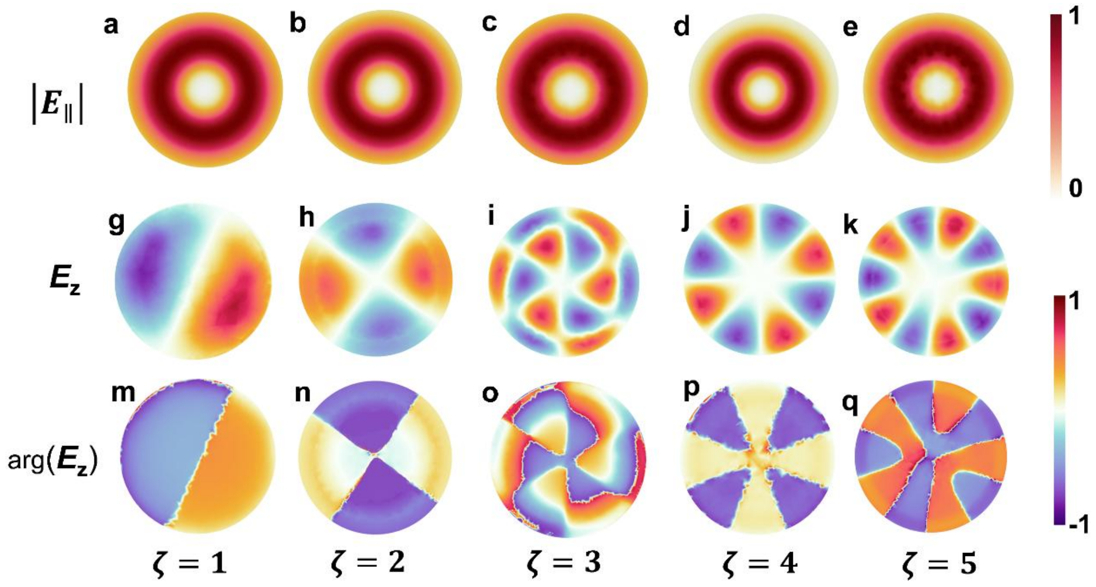

Figure S5. Far-field field distributions for SSH-ring modes with increasing winding number ζ=1-5. (a-e) Polarization-summed in-plane field amplitude $|E_{\parallel}|$ showing the expected annular (doughnut) profiles for all ζ. (g-k) Longitudinal field component Ez revealing the characteristic $2|\zeta|$-lobe (petal) structure that arises from tight focusing, spin-orbit coupling, and discrete azimuthal sampling. (m-q) Wrapped phase $\arg(Ez) \in (-\pi,\pi]$; the apparent $\pm\pi$ jumps are due to phase wrapping and nodal lines, not to the absence of OAM. Together these panels demonstrate that the beam carries OAM set by the SSH winding number while individual components can display petal-like patterns.

IV.3. Chiral Excitation of the SSH Ring

To excite the SSH ring, we define a chiral dipole excitation of the form:

$$|\psi_m\rangle = \frac{1}{\sqrt{2N}} \sum_{j=1}^{2N} e^{im\phi_j} |j\rangle \tag{30}$$

$\zeta \in \mathbb{Z}$: angular momentum of the emitter (e.g., $m = \pm 8$),

$\phi_j = 2\pi j/(2N)$: azimuthal angle of site j

$|j\rangle$: field localized at site j

This excitation profile simulates vortex beams (such as Laguerre-Gaussian modes) or circularly polarized dipoles localized above the ring. The projection of this excitation onto the SSH eigenmodes allows us to determine which modes are excited.

IV.4. Mode Projection and Spectral Selection

Let the eigenstates of $\hat{H}_{SSH}$ be $|\varphi_n\rangle$ with eigenvalues E_n. The probability that mode n is excited by a dipole of angular momentum m is [12]

$$P_n^{(m)} = |\langle\varphi_n|\varphi_m\rangle|^2 \tag{31}$$

By calculating $P_n^{(m)}$ for each eigenstate and plotting the corresponding energy spectrum E_n (as shown in Figure S6(a)) we observe a clear asymmetry in the spectral response depending on the excitation handedness. Specifically, counter-propagating dipolar excitation (shown in gray/black) selectively couples only to extended bulk modes, whereas co-propagating (chiral) excitation robustly addresses two spectrally isolated modes located within the bandgap (highlighted in red). These modes are unambiguously identified as topological bound states, consistent with theoretical predictions for chiral SSH systems where symmetry and directional excitation play a crucial role.

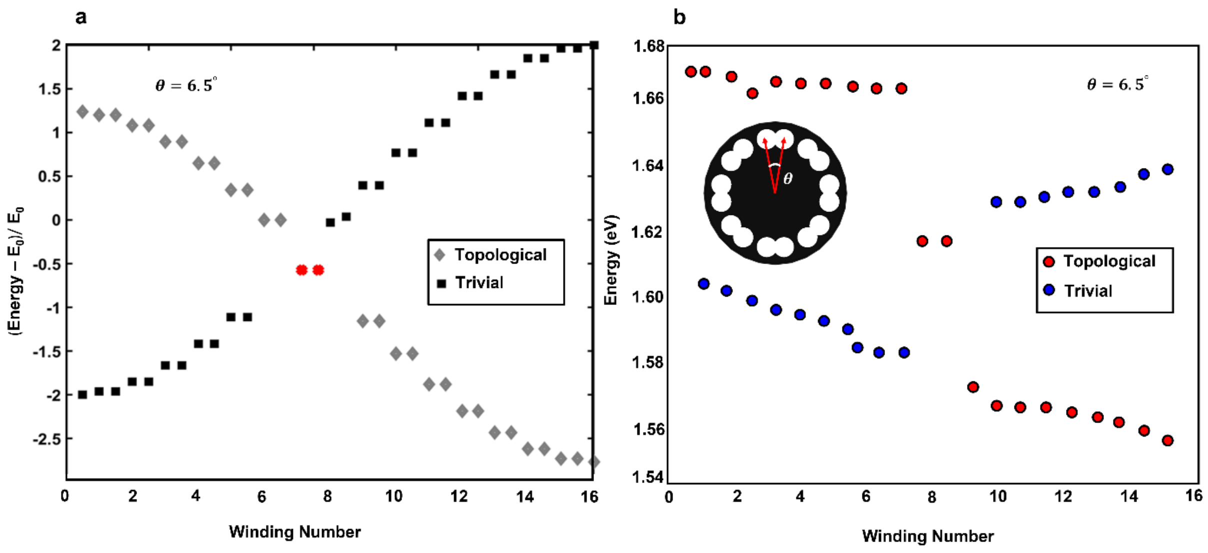

Figure S6: Topological mode selection in an SSH nanohole ring. Comparison of theoretical and numerical results for a Su–Schrieffer–Heeger (SSH) ring composed of 16 unit cells. Each unit cell consists of circular nanoholes (diameter D=248 nm) perforated into a 40 nm-thick gold disk with radius b=1380 nm. The left panel displays the theoretically calculated eigenvalue spectrum of the SSH tight-binding Hamiltonian, incorporating a dimerization parameter corresponding to a 6.5° angular modulation. Red markers highlight spectrally isolated topological edge modes that appear exclusively under co-propagating (chirality-matched) excitation. In contrast, counter-propagating excitation (gray/black) excites only extended bulk modes. The right panel shows eigenvalues of the SSH chain ring resonator for a range of loop numbers (ζ = 1–16). Topological modes were discerned for ζ equal to 8, with a dimerization parameter of 6.5° in a chain comprising 16 unit cells. The emergence of two distinct in-gap modes (topological bound states) is associated with the excitation of counter-rotating eigenmodes carrying angular momentum m=±ζ, reflecting the system's nontrivial winding number and chiral symmetry. The theoretical and simulated results together confirm robust spectral isolation and directional selectivity of topological modes in the plasmonic SSH ring.

The theoretically calculation and numerically simulation results clearly show that the topological states are well- separated from the bulk spectrum. Their spectral isolation makes them ideal candidates for robust chiral single-photon sources as the chiral topological bath, as they minimize undesired coupling to bulk modes. The emergence of two distinct topological modes within the revealed band gap, as depicted in Figure S3, can be attributed to the excitation of two counter-rotating circular modes ($m = \pm\zeta$). The manifestation of positive and negative winding numbers becomes apparent upon analysis of the orbital angular momentum associated with these modes.

This chiral mode selectivity, arising from the interplay of angular momentum and topology which provides a natural link to the many-emitter effective spin Hamiltonian. When the excitation is circularly polarized (e.g., σ^+), the induced dipole inherits spin angular momentum from the incident field, aligned with the optical field: $\boldsymbol{S} \propto \mathrm{Im}(\mathbf{E}^* \times \mathbf{E})$. In vortex-like SSH eigenmodes, this spin is transverse and locked to the orbital angular momentum (OAM) of the mode. Consequently, spin–OAM locking ensures that σ^+-polarized excitation preferentially couples to clockwise-propagating topological edge modes, while σ^- addresses counterclockwise modes. The resulting emitter–emitter interaction is effectively chiral and long-range, mediated by the nontrivial SSH bath. Within the single-excitation subspace the interaction Hamiltonian on Eq. (25) reduce to an effective spin-exchange model [13]:

$$H_{int} = \sum_{m<n} J_{mn}^{chiral} (\sigma_m^+ \sigma_n^- + h.c.) \tag{32}$$

where σ_m^+ and σ_n^- are raising/lowering operators for the m-th and n-th gold nanospheres, the interaction strength $J_{mn}^{chiral} \propto E_G^*(\mathbf{r}_m) E_G(\mathbf{r}_n)$ inherits both directionality and range from the vortex field profile and SSH topology. This establishes a direct connection between the topological photonic bath and emergent quantum magnetism among emitters, positioning the system as a promising platform for exploring

dissipative quantum many-body dynamics and chiral spin physics in a synthetic gauge field as we developed in the many-emitter effective spin formalism discussed in the main text.

Section V: Purcell-Enhanced Coupling and Far-Field Transfer by Gold Nanosphere

In our hybrid plasmonic–topological platform, gold nanospheres embedded in the SSH nanohole ring do not merely act as passive quantum emitters but function as active nanophotonic antennas that coherently interact with and re-emit structured topological modes. This interaction is governed by a rich interplay of Purcell-enhanced decay, local field overlap, and spin–orbit–momentum coupling.

V.1. Purcell Enhancement in the Topological Bath

The Purcell factor quantifies the modification of the spontaneous emission rate Γ of a dipole emitter due to its environment [14]:

$$F_P(r,\omega) = \frac{6\pi c}{\omega} \cdot \mathrm{Im}[d^* \cdot \mathbf{G}(\mathbf{r},\mathbf{r},\omega) \cdot \mathbf{d}] \tag{33}$$

where $\mathbf{d}$ is the dipole moment, and $\mathbf{G}$ is the Green's tensor for the SSH ring. In our system, $\mathbf{G}$ features sharp resonances due to topological edge states confined along the ring, strongly boosting Γ when the emitter frequency lies near midgap:

$$\Gamma_{\mathrm{eff}} = \Gamma_0 \cdot F_P \tag{34}$$

where Γ_0 is the vacuum decay rate. The Purcell factor diverges near band edges and reaches a maximum for mid-gap bound states, creating a localized enhancement channel for gold spheres. The total decay rate of a dipole near a metallic nanostructure (here, gold sphere) can be decomposed as:

$$\Gamma_{\mathrm{tot}} = \Gamma_{\mathrm{rad}} + \Gamma_{\mathrm{nrad}} \tag{35}$$

where Γ_{rad} and Γ_{nrad} represent power radiated to the far-field and energy dissipated in the metal, respectively. The efficiency of far-field transfer is governed by the quantum efficiency:

$$\eta = \frac{\Gamma_{\mathrm{rad}}}{\Gamma_{\mathrm{tot}}} \tag{36}$$

Due to the dipolar nature of the gold sphere resonance (diameter ≈ 60 nm), only the dipole plasmon mode couples effectively to the ring field. When aligned to the local field polarization of the SSH topological mode (e.g., via a σ^+ pump), the nanoparticle acts as a directional antenna with spin-selective emission.

V.2. Transferring the coherency to Far Field

The interaction strength between an emitter and a structured mode is given by the overlap integral [15]:

$$g(r_0) = \int d^3\mathbf{r}\, \mathbf{E}^*_{\mathrm{SSH}}(\mathbf{r}) \cdot \mathbf{E}_{\mathrm{NP}}(\mathbf{r} - \mathbf{r_0}) \tag{37}$$

where $\mathbf{r_0}$ is emitter position, $\mathbf{E}_{\mathrm{SSH}}$ and $\mathbf{E}_{\mathrm{NP}}$ are the electric field of the SSH topological mode and near-field of the gold nanoparticle dipole resonance. Only those nanoparticles that are placed at correct helicity of the bath couple efficiently. This explains the chiral directionality of the interaction and the nanoparticles coupling to the chiral path.

Upon coherent excitation, each gold nanosphere becomes a plasmonic nanoantenna radiating into free space with angular momentum inherited from the SSH mode: i. The azimuthal OAM ζ is mapped into the phase of the radiated beam, ii. The SAM (σ^+ or σ^-) maps to circular polarization in the far-field. The scattered field at large r follows:

$$\mathrm{E}_{far}(\theta,\phi) \propto \eta \cdot \mathbf{P} \cdot \frac{e^{ikr}}{r} \cdot e^{i\zeta\phi} \tag{38}$$

Thus, the coherent emitter-edge-mode system acts as a directional topological beam launcher, converting topological plasmonic modes into free-space modes with defined spin and orbital angular momentum.

VI. Flowchart of the training and searching process for the proposed algorithm[16].

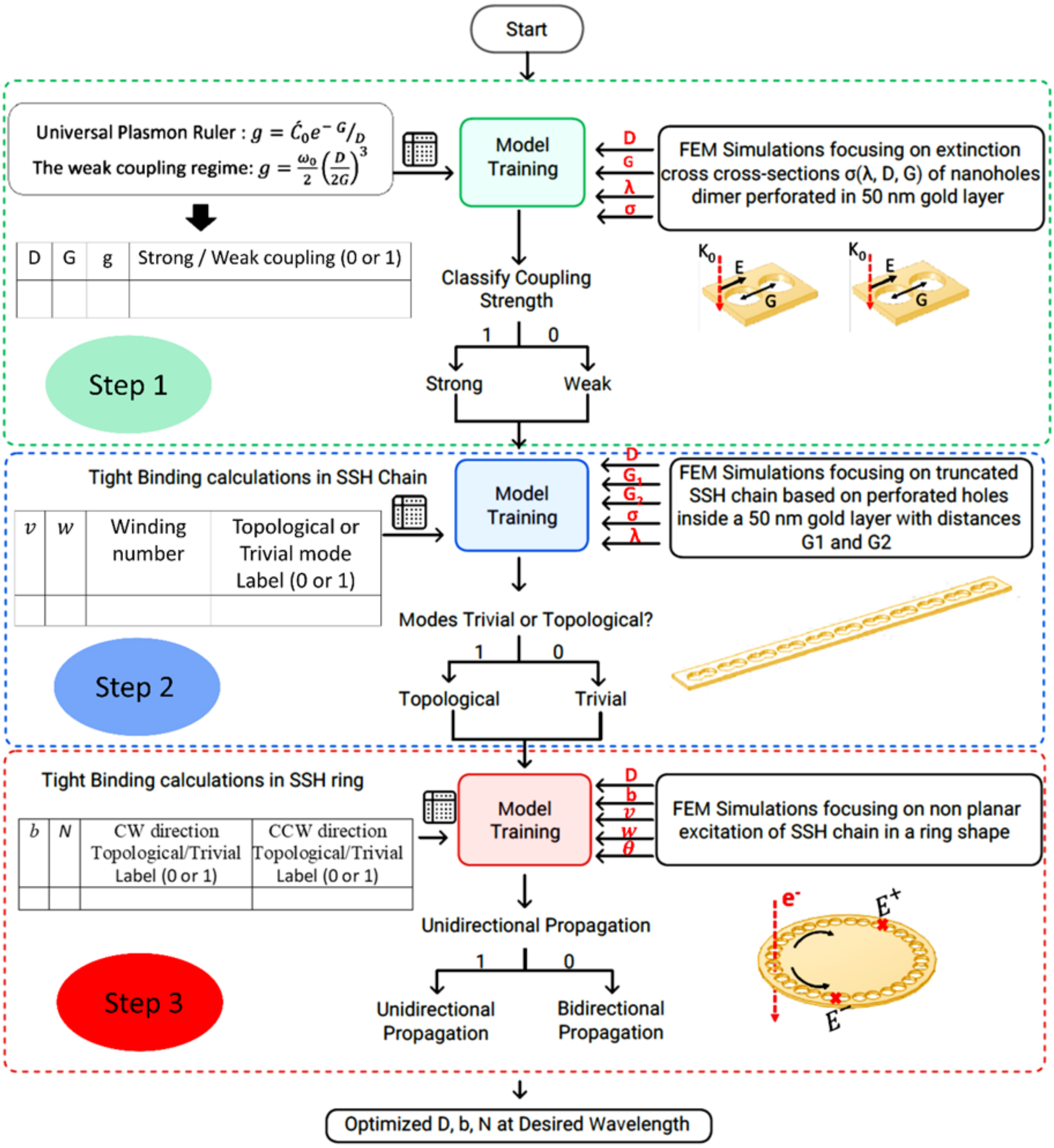

Figure S7: The process comprises three stages: (1) classification of coupling strength via finite-element method (FEM) simulations, using a labeled dataset informed by approximate analytical formulas for the strong- and weak-coupling regimes; (2) identification of topological and trivial modes in the SSH chain through combined tight-binding calculations and FEM simulations; and (3) detection of unidirectional propagation in an SSH ring under non-planar excitation. In each stage, simulation data guide model training to optimize the structural parameters D, G, b, and N for the target wavelength. The results confirm that the field profile remains aligned with the propagation direction in both cases, demonstrating the robustness of the topological mode [16].

References

1. Fujita M, Igami M, Nakada K. Lattice Distortion in Nanographite Ribbons. J Physical Soc Japan. 1997;66(7).

2. Jürß C, Bauer D. High-harmonic generation in Su-Schrieffer-Heeger chains. Phys Rev B. 2019;99(19).

3. Downing CA, Sturges TJ, Weick G, Stobińska M, Martín-Moreno L. Topological Phases of Polaritons in a Cavity Waveguide. Phys Rev Lett. 2019;123(21).

4. Davoodi F, Talebi N. Unidirectional Wave Propagation in a Topological Plasmonic Ring Resonator via a Symmetry-Broken Excitation Scheme. ACS Appl Nano Mater. 6(22):20823–30.

5. Bello M, Platero G, Cirac JI, González-Tudela A. Unconventional quantum optics in topological waveguide QED. Sci Adv. 2019;5(7).

6. Sirker J, Maiti M, Konstantinidis NP, Sedlmayr N. Boundary fidelity and entanglement in the symmetry protected topological phase of the SSH model. Journal of Statistical Mechanics: Theory and Experiment. 2014;2014(10).

7. Gardiner CW, Zoller P. Quantum noise: a handbook of Markovian and non-Markovian quantum stochastic methods with applications to quantum optics. Vol. 16, Springer series in synergetics,. 2004.

8. Kofman AG, Kurizki G, Sherman B. Spontaneous and induced atomic decay in photonic band structures. J Mod Opt. 1994;41(2).

9. Haroche S. Nobel Lecture: Controlling photons in a box and exploring the quantum to classical boundary. Rev Mod Phys. 2013;85(3).

10. Lodahl P, Mahmoodian S, Stobbe S. Interfacing single photons and single quantum dots with photonic nanostructures. Vol. 87, Reviews of Modern Physics. 2015.

11. Chang DE, Vuletić V, Lukin MD. Quantum nonlinear optics - Photon by photon. Nat Photonics. 2014;8(9).

12. Yao NY, Laumann CR, Gopalakrishnan S, Knap M, Müller M, Demler EA, et al. Many-body localization in dipolar systems. Phys Rev Lett. 2014;113(24).

13. Davoodi F. Beyond Decoherence: Control the Collective Quantum Dynamics of Quasi Particles in Topological Interface [Internet]. 2025 Jun [cited 2025 Jul 9]. Available from: https://arxiv.org/abs/2506.12805

14. Dung HT, Knoell L, Welsch DG. Three-dimensional quantization of the electromagnetic field in dispersive and absorbing inhomogeneous dielectrics. Phys Rev A. 1998;57(5).

15. Törmö P, Barnes WL. Strong coupling between surface plasmon polaritons and emitters: A review. Vol. 78, Reports on Progress in Physics. 2015.

16. Davoodi F. Active Physics-Informed Deep Learning: Surrogate Modeling for Nonplanar Wavefront Excitation of Topological Nanophotonic Devices. Nano Lett. 2025 Jan 15;25(2):768–75.
