# Supplementary figures and images for: From bound states to quantum spin models: chiral coherent dynamics in topological photonic rings
Source: Nanophotonics. 2025 Nov 25;14(24):4397–409. doi: 10.1515/nanoph-2025-0473 (PMC12704495; doi:10.1515/nanoph-2025-0473)

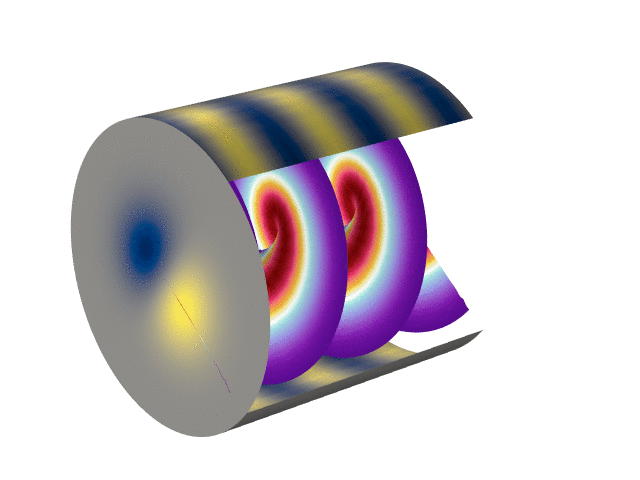

Supplement: Supplementary file 2 — Supplementary Material Details [file j_nanoph-2025-0473_suppl_002.gif]

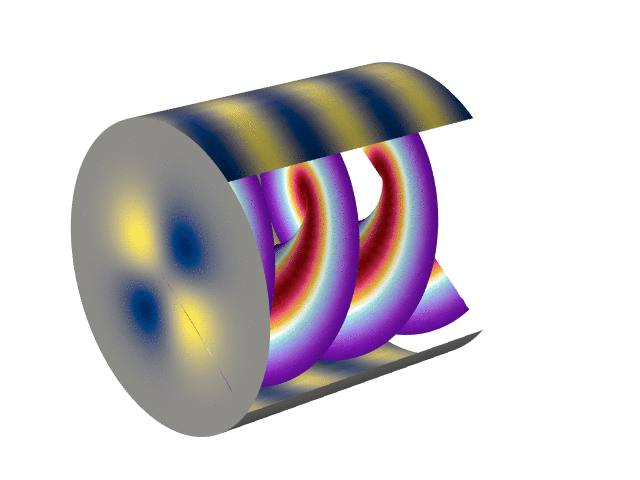

Supplement: Supplementary file 3 — Supplementary Material Details [file j_nanoph-2025-0473_suppl_003.gif]

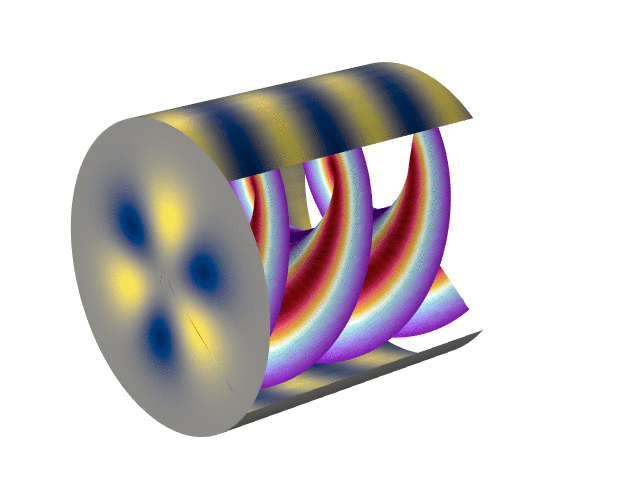

Supplement: Supplementary file 4 — Supplementary Material Details [file j_nanoph-2025-0473_suppl_004.gif]

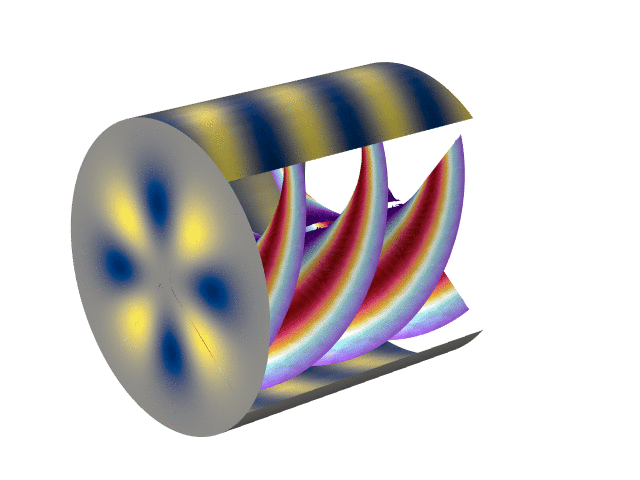

Supplement: Supplementary file 5 — Supplementary Material Details [file j_nanoph-2025-0473_suppl_005.gif]

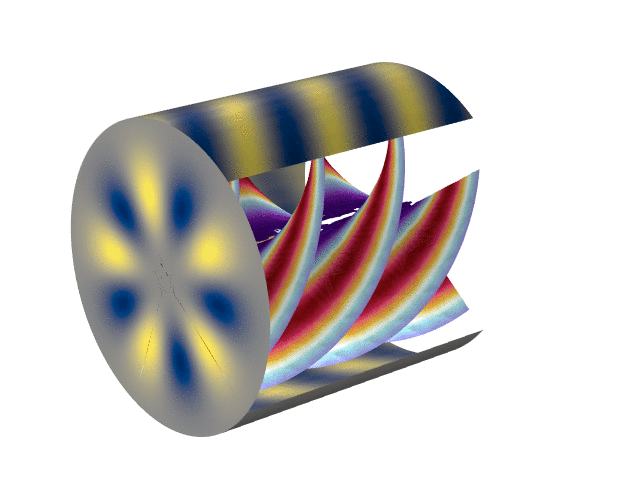

Supplement: Supplementary file 6 — Supplementary Material Details [file j_nanoph-2025-0473_suppl_006.gif]
